# DNA methylation and 28-year cardiovascular disease risk in type 1 diabetes: the Epidemiology of Diabetes Complications (EDC) cohort study

**DOI:** 10.1186/s13148-023-01539-0

**Published:** 2023-08-02

**Authors:** Rachel G. Miller, Josyf C. Mychaleckyj, Suna Onengut-Gumuscu, Eleanor Feingold, Trevor J. Orchard, Tina Costacou

**Affiliations:** 1 Department of Epidemiology, University of Pittsburgh, 130 N. Bellefield Avenue, Suite 339, Pittsburgh, PA 15213 USA; 2 Center for Public Health Genomics, University of Virginia, Charlottesville, VA USA; 3 Department of Human Genetics, University of Pittsburgh, Pittsburgh, PA USA

**Keywords:** Type 1 diabetes, Epigenetics, DNA methylation, Cardiovascular disease, Coronary artery disease, Major adverse cardiovascular events, HbA1c, Glycemic exposure

## Abstract

**Background:**

The potential for DNA methylation (DNAm) as an early marker for cardiovascular disease (CVD) and how such an association might differ by glycemic exposure has not been examined in type 1 diabetes, a population at increased CVD risk. We thus performed a prospective epigenome-wide association study of blood leukocyte DNAm (EPIC array) and time to CVD incidence over 28 years in a childhood-onset (< 17 years) type 1 diabetes cohort, the Pittsburgh Epidemiology of Diabetes Complications (EDC) study (*n* = 368 with DNA and no CVD at baseline), both overall and separately by glycemic exposure, as measured by HbA1c at baseline (split at the median: < 8.9% and ≥ 8.9%). We also assessed whether DNAm-CVD associations were independent of established cardiometabolic risk factors, including body mass index, estimated glucose disposal rate, cholesterol, triglycerides, blood pressure, pulse rate, albumin excretion rate, and estimated glomerular filtration rate.

**Results:**

CVD (first instance of CVD death, myocardial infarction, coronary revascularization, ischemic ECG, angina, or stroke) developed in 172 participants (46.7%) over 28 years. Overall, in Cox regression models for time to CVD, none of the 683,597 CpGs examined reached significance at a false discovery rate (FDR) ≤ 0.05. In participants with HbA1c < 8.9% (*n* = 180), again none reached FDR ≤ 0.05, but three were associated at the a priori nominal significance level FDR ≤ 0.10: cg07147033 in *MIB2*, cg12324048 (intergenic, chromosome 3), and cg15883830 (intergenic, chromosome 1). In participants with HbA1c ≥ 8.9% (n = 188), two CpGs in loci involved in calcium channel activity were significantly associated with CVD (FDR ≤ 0.05): cg21823999 in *GPM6A* and cg23621817 in *CHRNA9*; four additional CpGs were nominally associated (FDR ≤ 0.10). In participants with HbA1c ≥ 8.9%, DNAm-CVD associations were only modestly attenuated after cardiometabolic risk factor adjustment, while attenuation was greater in those with HbA1c < 8.9%. No pathways were enriched in those with HbA1c < 8.9%, while pathways for calcium channel activity and integral component of synaptic membrane were significantly enriched in those with HbA1c ≥ 8.9%.

**Conclusions:**

These results provide novel evidence that DNAm at loci involved in calcium channel activity and development may contribute to long-term CVD risk beyond known risk factors in type 1 diabetes, particularly in individuals with greater glycemic exposure, warranting further study.

**Supplementary Information:**

The online version contains supplementary material available at 10.1186/s13148-023-01539-0.

## Background

Despite advances in treatment, people with type 1 diabetes continue to be at dramatically increased risk of cardiovascular disease (CVD) compared to people without diabetes [1]. Yet, no combination of genetic or clinical risk factors, including glycemic control and traditional cardiometabolic risk factors, fully explain the increased CVD risk associated with type 1 diabetes [2, 3]. Thus, it is likely that complex interactions between genetic factors and glycemic exposure, as well as other environmental and lifestyle exposures, influence earlier development of atherosclerosis in type 1 diabetes. DNA methylation (DNAm) provides a link between genetic susceptibility and risk factor exposure and its study has potential to uncover novel pathways to development of complex phenotypes, including CVD. Studies in the general population have shown differential patterns of methylation are associated with atherosclerosis, hypertension, lipids, and inflammation and such differential methylation may modify risk of clinical CVD events through those mechanisms [4]. Furthermore, a meta-analysis from the Cohorts for Heart and Aging Genetic Epidemiology (CHARGE) consortium identified 52 CpGs where DNAm was prospectively associated with incidence of coronary heart disease over a mean follow-up of 11 years [5]. However, to date there have been no published studies on methylation and CVD in type 1 diabetes despite the increased CVD burden carried by this population.

In type 1 diabetes, DNAm has been associated with glycemic exposure, as measured by HbA1c, in both the Diabetes Control and Complications Trial (DCCT)/ Epidemiology of Diabetes Interventions and Complications (EDIC) [6] and the Pittsburgh Epidemiology of Diabetes Complications (EDC) study [7]. In DCCT/EDIC, methylation at HbA1c-associated CpG sites explained much of the statistical association between HbA1c and risk of future microvascular disease, including proliferative retinopathy and diabetic kidney disease (DKD) [6]. Those results provide the strongest evidence thus far that past glycemic exposure is related to differential methylation, which may in turn influence future diabetes complication risk. A few other studies in type 1 diabetes have found associations between DNAm and proliferative retinopathy [8] and DKD [9–12] but those studies have been almost exclusively cross sectional and have not addressed differences in associations by glycemic control. Thus, given the limited prospective data on DNAm and diabetes complications in general and the lack of data on DNAm and CVD in type 1 diabetes specifically, our objective was to perform a prospective epigenome-wide association study (EWAS) of the association between whole blood-derived leukocyte DNAm and subsequent incidence of CVD over 28 years in type 1 diabetes. We hypothesized there are loci with DNAm associated with time-to-first CVD event in the Pittsburgh EDC study, a childhood-onset type 1 diabetes cohort. Additionally, as we have recently demonstrated risk factors for CVD differ by level of glycemic exposure [13], we further hypothesized that CVD-associated DNAm would differ by baseline HbA1c.

## Results

**Fig. 1:**
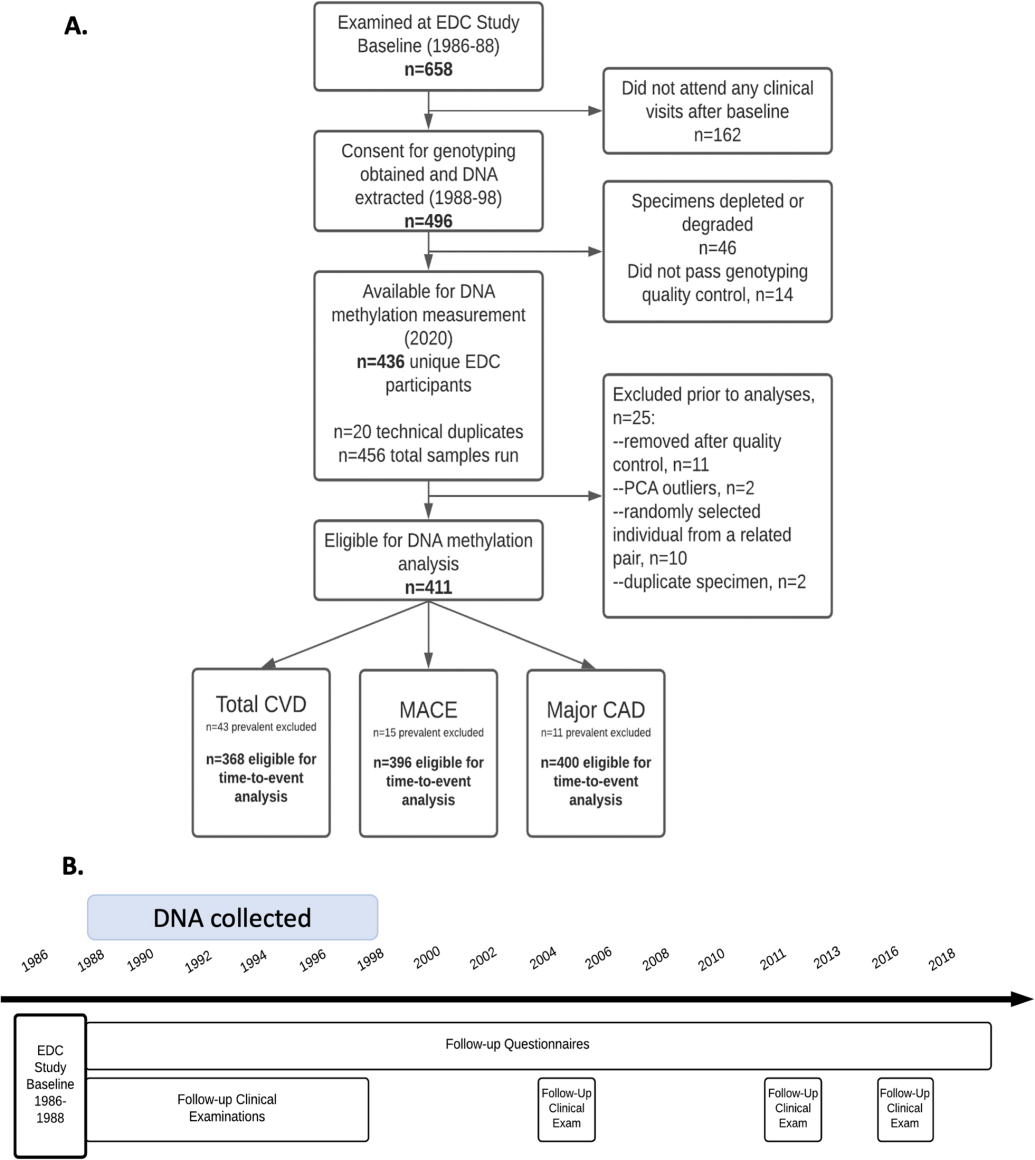
Flowchart and timeline for the EDC DNA methylation sub-study. **A** Flowchart summarizing the determination of the analytic sample for DNA methylation and time to cardiovascular disease event in the EDC type 1 diabetes cohort. CVD = cardiovascular disease, MACE = major adverse cardiovascular event, CAD = coronary artery disease. **B** Study timeline. “Baseline” for the current analysis is the participant-specific time point at which the DNA specimen was collected

Of the 368 individuals eligible for the primary analysis (Fig. 1), 172 (46.7%) developed CVD by 28 years. For the secondary outcomes, 28.0% (111 of 396 individuals) developed major adverse cardiovascular events (MACE) and 25.5% (102 of 400 individuals) major coronary artery disease (CAD). Kaplan Meier curves for time to total CVD, MACE, and major CAD over the 28-year follow-up are shown in Additional file 1: Fig. S2. Baseline characteristics overall and by total CVD incidence status are in Table 1.

**Table 1:** Baseline characteristics of members of the Epidemiology of Diabetes Complications (EDC) Study DNA methylation sub-cohort who were free of any CVD at baseline

Characteristic	Overall (*n* = 368)	Incident CVD (*n* = 172)	No CVD (*n* = 196)	*p* value‡
Age, years	29.2 (7.7)	32.2 (7.4)	26.5 (7.0)	< 0.001
Type 1 diabetes duration, years	20.9 (7.6)	24.0 (7.8)	18.1 (6.3)	< 0.001
Age at type 1 diabetes Onset, years	8.3 (4.1)	8.2 (3.8)	8.3 (4.3)	0.927
Female sex, % (*n*)	46.3% (176)	50.0% (86)	45.9% (90)	0.581
≥ Bachelor’s degree, % (*n*)	35.3% (130)	29.7% (51)	40.3% (79)	0.003
HbA1c, %	9.0 (1.5)	9.3 (1.5)	8.9 (1.5)	0.007
HbA1c, mmol/mol	75.3 (16.4)	77.7 (16.6)	73.2 (16.0)	
*Smoking status, % (n)*				
Never	63.9% (235)	58.1% (100)	68.9% (135)	0.001
Past	15.5% (57)	15.7% (27)	15.3% (30)	
Current	20.7% (76)	26.2% (45)	15.8% (31)	
Smoking, pack-years*	0 (0, 3)	0 (0, 10)	0 (0, 0.2)	< 0.001
Body Mass Index, kg/m^2^	24.2 (3.3)	24.8 (3.3)	23.7 (3.2)	< 0.001
Insulin dose, insulin units/kg body weight	0.78 (0.25)	0.77 (0.25)	0.79 (0.24)	0.122
MDI^†^ or insulin pump use, % (*n*)	13.8% (50)	13.0% (22)	14.5% (28)	0.437
Self-monitoring of blood glucose, % (*n*)	72.6% (267)	72.0% (124)	73.0% (143)	0.925
Estimated glucose disposal rate, mg/kg/min	7.55 (2.01)	6.95 (2.14)	8.08 (1.74)	< 0.001
Total cholesterol (mg/dl)	186.8 (40.5)	202.4 (38.6)	173.1 (37.1)	< .0001
HDLc (mg/dl)	53.4 (12.4)	53.5 (12.7)	53.4 (12.3)	0.774
Non-HDLc (mg/dl)	133.4 (39.1)	148.9 (38.3)	119.7 (34.8)	< 0.001
Triglycerides (mg/dl)*	80.0 (57.0, 118.0)	95.5 (67.8, 137.3)	67.0 (53.0, 118.0)	< 0.001
Lipid medication, % (*n*)	1.1% (4)	2.3% (4)	0% (0)	0.008
Systolic blood pressure (mmHg)	113.8 (15.8)	118.1 (17.6)	110.0 (12.9)	< 0.001
Diastolic blood pressure (mmHg)	72.5 (11.0)	74.5 (12.1)	70.7 (9.5)	< 0.001
Hypertension, % (*n*)	17.4% (64)	26.7% (46)	9.2% (18)	< 0.001
Blood pressure medication, % (*n*)	11.0% (40)	18.0% (31)	4.6% (9)	< 0.001
Pulse rate, bpm	75.7 (10.8)	77.4 (10.5)	74.2 (10.8)	0.005
Albumin excretion rate, µg/min*	11.4 (5.5, 22.9)	19.6 (8.1, 288.5)	8.9 (5.5, 73.9)	< 0.001
Estimated glomerular filtration rate, ml/min/1.73 m^2^	112.7 (31.1)	106.7 (32.4)	117.9 (28.9)	< 0.001
White blood cell count, × 10^9^ cells/l	6.9 (2.1)	7.3 (2.0)	6.7 (7.3)	< 0.001

Values are mean (SD) unless specified. *median (p25, p75), ^†^Multiple Daily Injections (≥ 3 insulin injections per day), ‡*p* value for the unadjusted association between each risk factor and CVD incidence from Cox regression

### Total CVD, MACE, and major CAD EWAS: overall cohort

There were no significant associations (false discovery rate, FDR ≤ 0.05) in the overall cohort (Additional file 1: Fig. S3); three CpGs had FDR ≤ 0.20 and were included in post hoc functional analyses: cg02768721, *PTPRN2* (*p* = 4.10 × 10^–7^), cg14524754, *B3GNTL1* (*p* = 6.21 × 10^–7^), and cg06648759, intergenic CpG, Chr 13 (*p* = 8.27 × 10^–7^). The ten most significant CpGs for total CVD are shown in Table 2. A QQ plot is shown in Additional file 1: Fig. S4 (traditional genomic inflation *λ* = 0.91). For the secondary outcomes, MACE and major CAD, again, no CpGs were significantly associated with either outcome (Additional file 1: Table S1). Three CpGs with FDR ≤ 0.20 for MACE and 21 CpGs with FDR ≤ 0.20 for major CAD were included in post hoc analyses. Using the traditional genomic inflation factor, EWAS for MACE (*λ* = 1.44) and major CAD (*λ* = 1.51) both had evidence of significant inflation. After *bacon* correction, inflation was reduced for both outcomes (*λ*.bacon = 1.13 and 0.87, respectively).

**Table 2:** Ten most significant (by p value) CpGs with DNA methylation associated with time to incident cardiovascular disease in the overall EDC cohort (n = 368)

Outcome	CpG	Chromosome	Location	CpG position (hg38)	Gene symbol	Location relative to gene	Log hazard ratio per 5% methylation	Standard error	*p* value	False discovery rate
Total CVD	cg02768721	7	S_Shore	158428677	*PTPRN2*	Body	1.610	0.318	4.10E−07	0.19
	cg14524754	17	N_Shelf	82967226	*B3GNTL1*	Body	0.571	0.115	6.21E−07	0.19
	cg06648759	13	OpenSea	40318613	(Intergenic)	n/a	0.564	0.114	8.27E−07	0.19
	cg08468449	1	OpenSea	25581110	(Intergenic)	n/a	0.838	0.174	1.40E−06	0.24
	cg04219688	19	OpenSea	2410500	TMPRSS9	Body	− 0.917	0.192	1.86E−06	0.25
	cg00474854	18	N_Shore	11821776	GNAL	Body	− 1.583	0.348	5.55E−06	0.44
	cg16992008	3	OpenSea	45920374	FYCO1	3'UTR	3.849	0.852	6.17E−06	0.44
	cg14499274	9	OpenSea	133840275	VAV2	Body	0.390	0.086	6.21E−06	0.44
	cg21571162	6	S_Shelf	33251876	VPS52	Body	0.993	0.222	7.48E−06	0.44
	cg05295851	1	S_Shore	9731967	CLSTN1	Body	− 3.511	0.789	8.59E−06	0.44

### HbA1c-stratified EWAS and cardiometabolic risk factor analysis

**Fig. 2:**
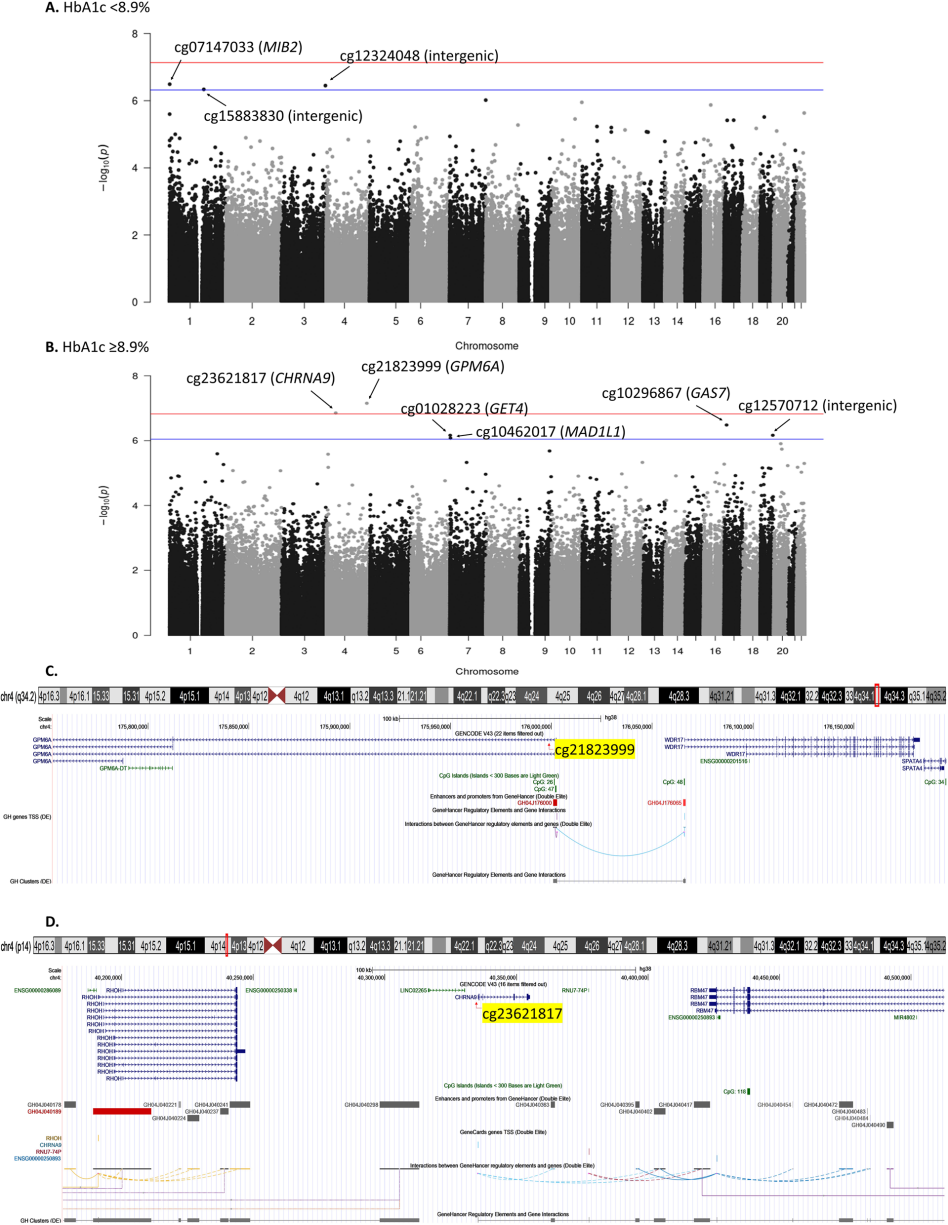
Manhattan plots for the HbA1c-stratified epigenome-wide associations of DNA methylation with time-to-CVD event (**A**, **B**) and gene track plots for CpGs with FDR ≤ 0.05 in those with HbA1c ≥ 8.9% (cg21823999, **C**; cg23621817 **D**). On Manhattan plots, red line indicates the significance level FDR ≤ 0.05; blue line indicates nominal significance level FDR ≤ 0.10

To assess our hypothesis that CVD-associated DNAm differs by HbA1c, we performed full EWAS stratified by median HbA1c at baseline (8.9%, 74 mmol/mol). Manhattan plots and gene track plots for CpGs with FDR ≤ 0.05 are in Fig. 2. In the *n* = 180 participants with HbA1c < 8.9%, 77 developed CVD (42.8%). No CpGs reached significance for CVD at FDR ≤ 0.05, but three were associated at the a priori nominal significance level (FDR ≤ 0.10) (Table 3). In the *n* = 188 participants with HbA1c ≥ 8.9%, 95 developed CVD (50.5%) and two CpGs were significantly associated with time to CVD (Table 3): cg21823999 in *GPM6A* (*p* = 7.03 × 10^–8^, FDR = 0.05) and cg23621817 in *CHRNA9* (*p* = 1.40 × 10^–7^, FDR = 0.05) (Table 3). As shown in Fig. 2, panel C, cg21823999 is in the promotor/enhancer region GH04J176000 (chr4: 176000933–176002800) and interacts with *WDR17.* Four additional CpGs had nominally suggestive associations (FDR ≤ 0.10) in those with HbA1c ≥ 8.9% (Table 3).

**Table 3:** CpGs with DNA methylation associated with time to total CVD incidence in the EDC study with false discovery rate ≤ 0.20 by median HbA1c at baseline

Subgroup	CpG	Chromosome	Location	CpG position (hg38)	Gene symbol	Location relative to gene	Log hazard ratio per 5% methylation	Standard error	*p* value	False discovery rate
HbA1c < 8.9%	**cg07147033**	**1**	**N_Shore**	**1614234**	***MIB2***	**TSS1500**	**0.828**	**0.162**	**3.23E−07**	**0.10**
	**cg12324048**	**3**	**OpenSea**	**194105335**	**(intergenic)**	**n/a**	**0.936**	**0.184**	**3.55E−07**	**0.10**
	**cg15883830**	**1**	**OpenSea**	**151495627**	**(intergenic)**	**n/a**	**3.170**	**0.629**	**4.60E−07**	**0.10**
	cg02768721	7	S_Shore	158428677	*PTPRN2*	Body	2.307	0.471	9.61E−07	0.15
	cg16943126	10	OpenSea	132497609	(intergenic)	n/a	1.101	0.226	1.12E−06	0.15
	cg09983897	16	Island	31471963	*TGFB1I1*	TSS1500; TSS200	6.928	1.433	1.34E−06	0.15
HbA1c ≥ 8.9%	**cg21823999**	**4**	**Island**	**176001929**	***GPM6A***	**5'UTR; Body**	**6.565**	**1.218**	**7.03E−08**	**0.05**
	**cg23621817**	**4**	**OpenSea**	**40335835**	***CHRNA9***	**Body**	**− 8.608**	**1.635**	**1.40E−07**	**0.05**
	**cg10296867**	**17**	**OpenSea**	**10145671**	***GAS7***	**Body**	**1.486**	**0.291**	**3.29E−07**	**0.07**
	**cg12570712**	**19**	**Island**	**53909630**	**(intergenic)**	**n/a**	**− 1.795**	**0.362**	**6.83E−07**	**0.09**
	**cg01028223**	**7**	**N_Shore**	**875511**	***GET4***	**TSS1500**	**− 4.048**	**0.815**	**6.85E−07**	**0.09**
	**cg10462017**	**7**	**N_Shelf**	**2144044**	***MAD1L1***	**Body**	**− 4.224**	**0.858**	**8.38E−07**	**0.09**
	cg17155697	20	S_Shelf	31852835	(intergenic)	n/a	− 3.436	0.709	1.24E−06	0.12
	cg22853588	20	S_Shore	36541477	*MYL9*	TSS200	0.486	0.102	1.83E−06	0.16
	cg08630171	9	OpenSea	129750961	*PTGES*	Body	4.168	0.878	2.08E−06	0.16
	cg18591304	1	OpenSea	209428437	*MIR205HG*	TSS1500	− 3.347	0.711	2.55E−06	0.16
	cg20136354	4	N_Shore	6663599	(intergenic)	n/a	0.540	0.115	2.63E−06	0.16

Bolded text indicates CpGs considered at least nominally suggestive (FDR ≤ 0.10)

**Fig. 3:**
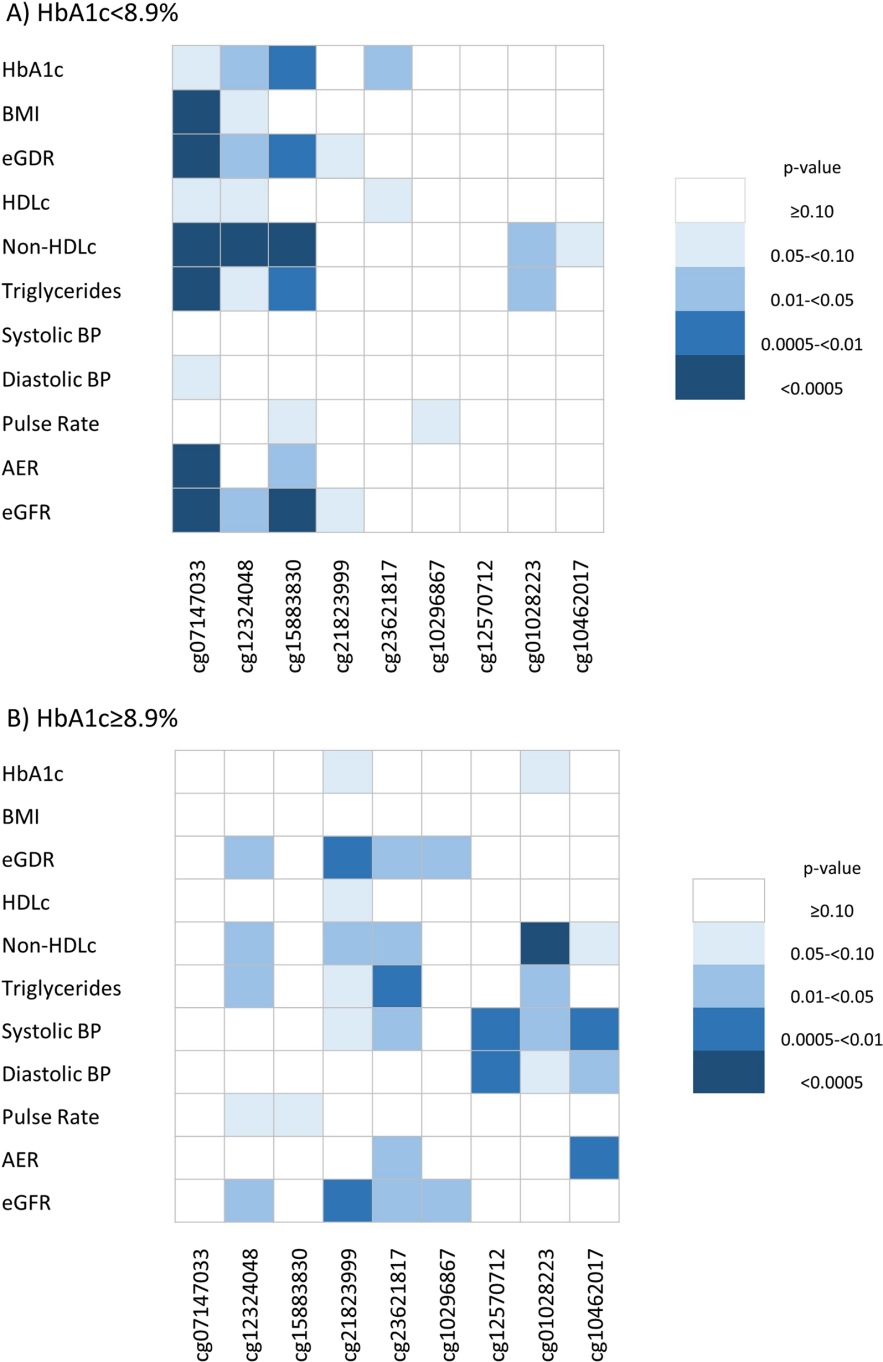
Heat maps of cardiometabolic phenotype associations with CVD-associated CpGs (FDR ≤ 0.10) by median baseline HbA1c

**Fig. 4:**
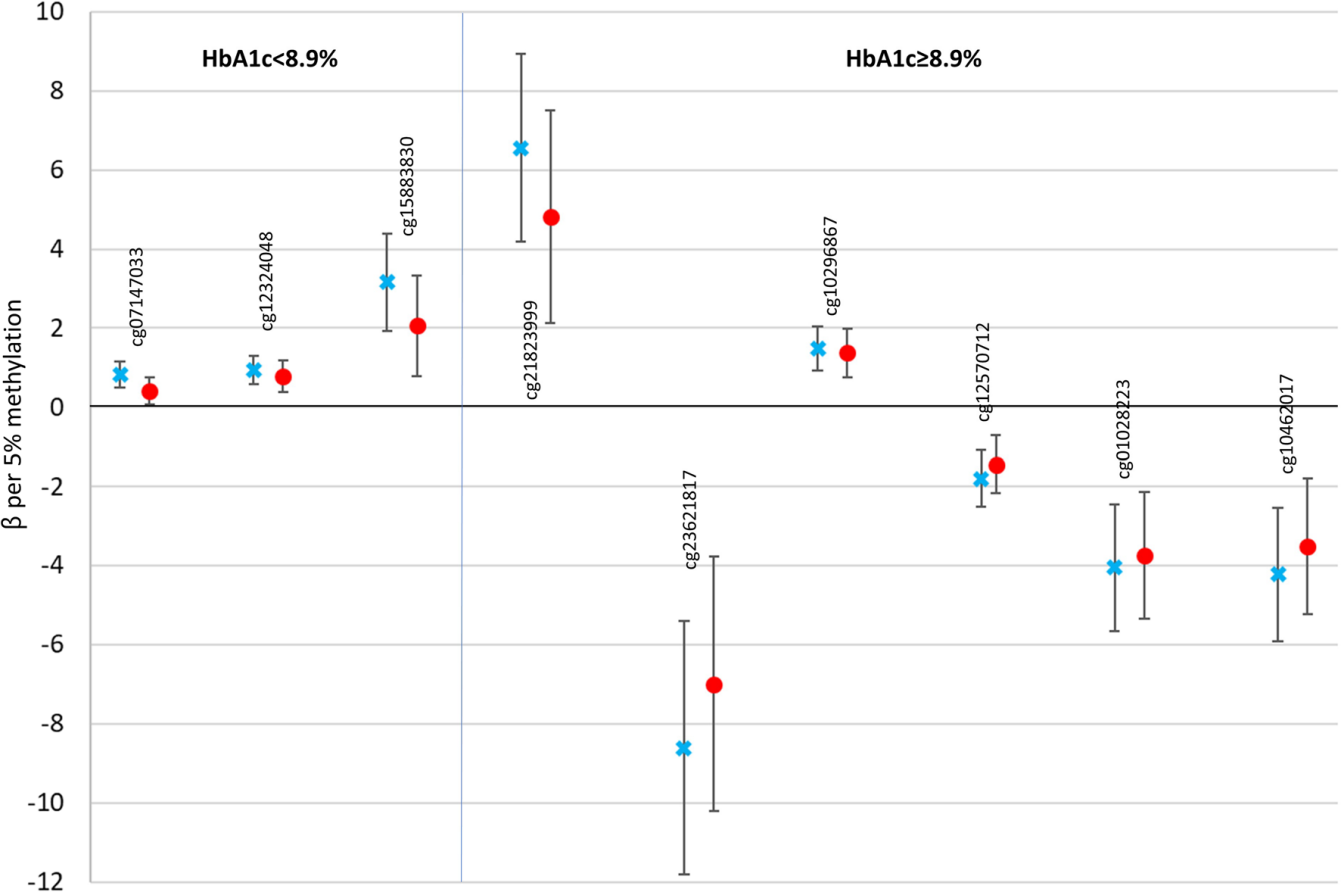
Log hazard ratio (*β*) per 5% methylation at each CpG associated with total CVD, unadjusted (blue x) and adjusted (red circles) for traditional cardiometabolic risk factors (error bars are 95% confidence limits) by baseline HbA1c

We next examined cross sectional associations between CVD-associated CpGs and established cardiometabolic risk factors separately by HbA1c at study baseline, when all participants were free of CVD, to gain insight into pathophysiologic pathways by lower or higher glycemic exposure. Heat maps of cardiometabolic risk factor associations with suggestive CVD-associated CpGs (FDR ≤ 0.10) by HbA1c are in Fig. 3 (Panel A: HbA1c < 8.9%, Panel B: HbA1c ≥ 8.9%). Regression coefficients for the same associations are in Additional file 1: Table S3 (HbA1c < 8.9%) and Additional file 1: Table S4 (HbA1c ≥ 8.9%). In those with HbA1c < 8.9%, greater DNAm at cg07147033 in *MIB2* was associated with a generally worse cardiometabolic risk factor pattern and there were significant (*p* < 0.0005) associations with BMI, eGDR, non-HDLc, triglycerides, AER, and eGFR. Also in those with HbA1c < 8.9%, greater DNAm at cg12323048 was significantly associated with higher non-HDLc and greater DNAm at cg15883830 was associated with higher non-HDLc and lower eGFR. In participants with HbA1c ≥ 8.9%, greater DNAm at cg01028223 (*GET4*) was significantly associated with lower non-HDLc only. After adjusting for cardiometabolic risk factors, associations between DNAm and time-to-CVD were attenuated to varying degrees (Fig. 4, Additional file 1: Table S5). Attenuation was greater in those with HbA1c < 8.9%, with 16–51% reduction in effect size after risk factor adjustment, compared to 7.5–26% reduction in those with HbA1c ≥ 8.9%.

### Methylation quantitative trait loci (meQTLs)

**Fig. 5:**
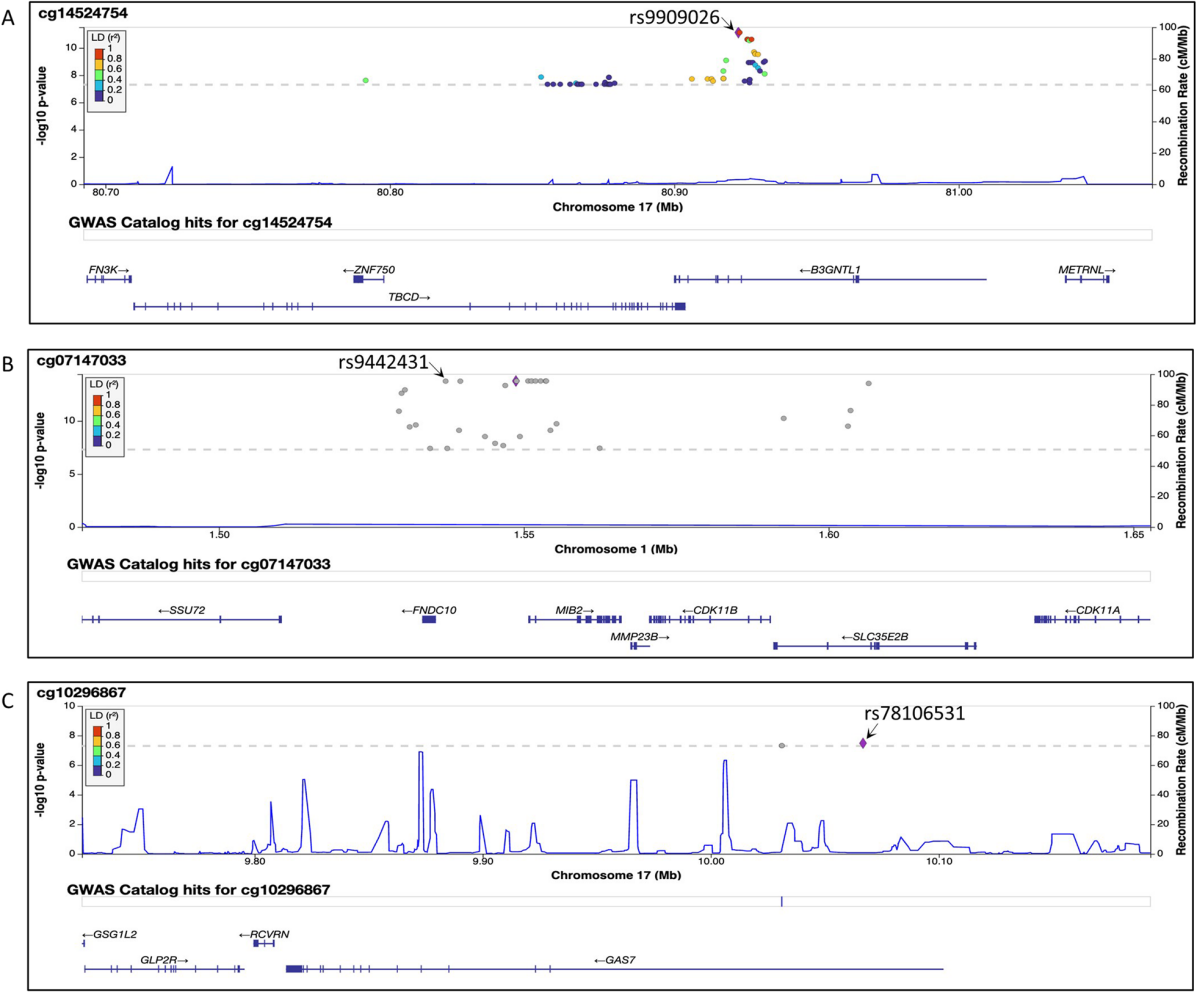
Locus Zoom plots for significant meQTLs for CpGs with DNAm associated with total CVD at FDR ≤ 0.20 in the EDC study. Each dot on the plot corresponds to a significant meQTL for DNAm at the corresponding CpG. The representative meQTLs are indicated on each plot

To avoid overlooking potentially important functional relationships, we applied a more liberal significance cut-off of FDR ≤ 0.20 to select CpGs for inclusion in post hoc functional analyses. We performed GWAS using genotyping with imputation to identify variants associated with methylation of the CVD-associated CpGs with FDR ≤ 0.20. We identified 59 cis variants in the *TBCD*–*B3GNTL1* region of chromosome 17 associated with cg14524754 methylation (Additional file 1: Table S6, Fig. S5) at genome-wide significance (*p* < 5 × 10^–8^); all 59 variants were confirmed to be significant meQTLs for cg14524754 in GoDMC (*p* < 5 × 10^–8^) and 29 were annotated as whole blood eQTLs (*p* < 0.0005) in GTEx. There were 31 cis variants in the *FNDC10–MIB2–CDK11B–SLC35E2B* region of chromosome 1 associated with cg07147033 methylation (Fig. 5); 20 were confirmed to be meQTLs for cg07147033 in GoDMC (*p* < 5 × 10^–8^) and 11 were whole blood eQTLs (*p* < 0.0005) in GTEx. Finally, there were 3 cis meQTLs for cg10296867 in *GAS7* (Fig. 5); all 3 were meQTLs in GoDMC (*p* < 5 × 10^–8^), but none were eQTLs in GTEx. No significant meQTLs were detected for any of the other CpGs with FDR ≤ 0.20 for total CVD in EDC.

For each of the three CpGs with significant meQTLs, we selected the variant with the strongest statistical association as the representative meQTL to estimate its association with CVD risk. The representative variants were rs9909026 (*B3GNTL1)* for cg14524754, rs9442431 (*MIB2)* for cg07147033, and rs78106531 (*GAS7)* for cg10296867. None were associated with CVD risk overall or in those with HbA1c ≥ 8.9% at baseline; however, rs9442431 (T > C) in *MIB2* was associated with CVD incidence in participants with HbA1c < 8.9% (HR = 2.20, 95% confidence interval: 1.44, 3.40, *p* = 0.00025).

### Network and gene set enrichment analysis (GSEA)

To gain insight into potential pathways underlying DNAm-CVD associations, we performed GSEA of gene ontology (GO) terms [14] and KEGG pathways [15] and identified a Reactome Functional Interaction (FI) network [16]. In GSEA, in both the overall cohort and in the subset with HbA1c < 8.9%, there were no significantly enriched GO terms or KEGG pathways including ≥ 2 loci. For the subset with HbA1c ≥ 8.9%, GO terms *calcium channel activity* (GO: 0005262, *p* = 0.0015) and *integral component of synaptic membrane* (GO: 0099699, *p* = 0.0015), which both include *GPM6A* and *CHRNA9, were* enriched (Additional file 1: Table S7). There were no enriched KEGG pathways including ≥ 2 loci.

**Fig. 6:**
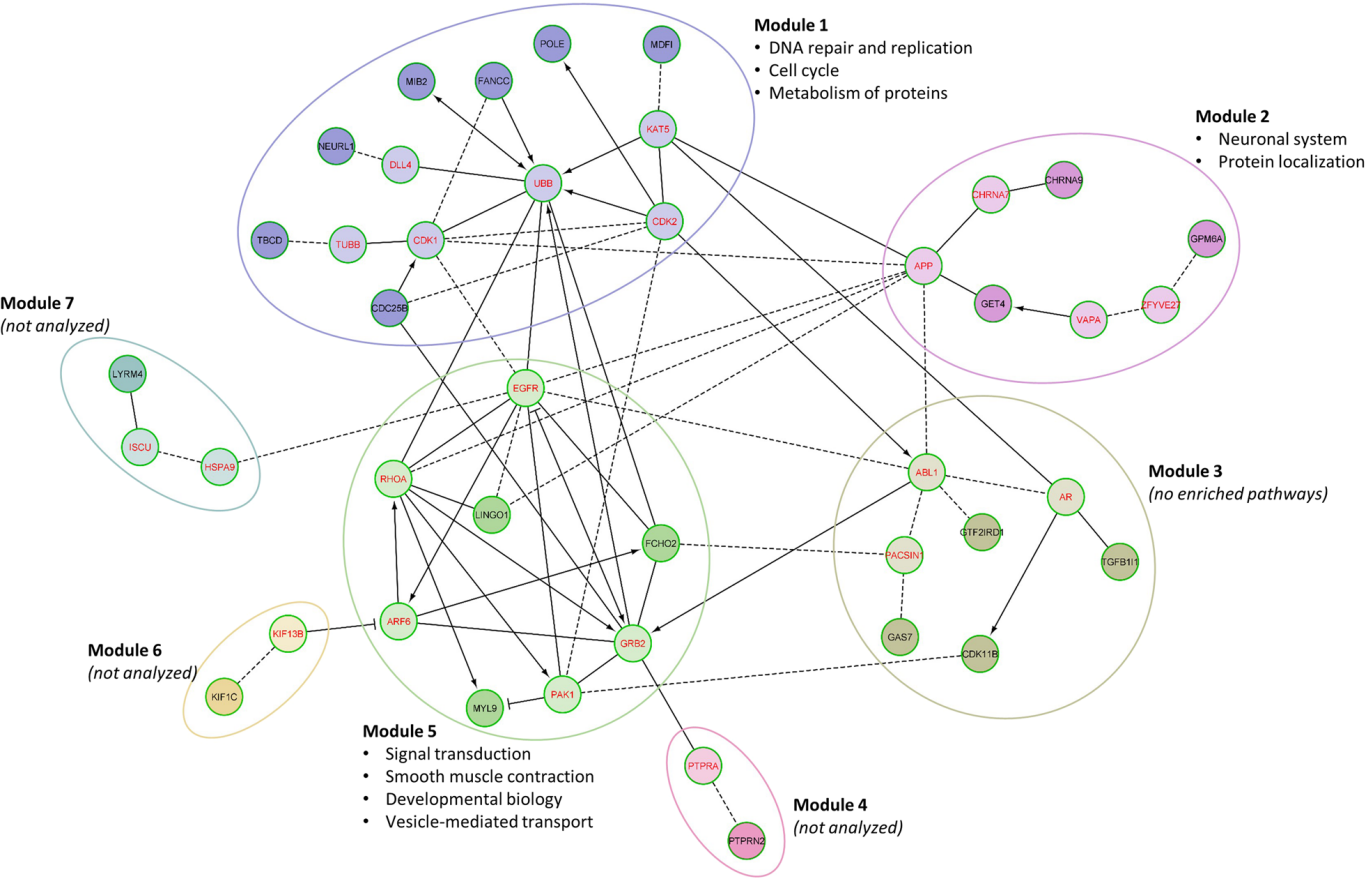
Reactome functional interaction network derived from the list of loci with DNA methylation associated with 28-year CVD incidence at FDR ≤ 0.20 in the EDC cohort. Candidate loci are indicated in black font (red font indicates linker genes used only to construct the network). Solid line = involved in same reaction as inputs or are components of a shared complex, $\to$=activator or catalyst, –|= inhibitor, dashed line = predicted interaction. Only modules with ≥ 2 candidate loci were analyzed for pathway enrichment

The candidate loci included in Reactome FI network analysis are listed in Additional file 1: Table S8. A FI network comprising seven modules was identified, three of which had significant pathway enrichment (Fig. 6). Significantly enriched top-level pathways (Additional file 1: Table S9) included *DNA repair, DNA replication, Cell cycle, and Metabolism of proteins* in Module 1 (*FANCC, POLE, TBCD*); *Neuronal system* and *Protein localization* in Module 2 (*CHRNA9, GET4*); and *Signal transduction, Smooth muscle contraction, Developmental biology, and Vesicle-mediated transport* in Module 5 (*LINGO1, FCHO2, MYL9*).

## Discussion

In this EWAS examining DNAm and 28-year CVD risk in type 1 diabetes, we identified several CVD-associated CpGs in loci across the genome, including sites in *GPM6A*, *CHRNA9*, *GAS7*, *GET4*, *MAD1L1,* and *MIB2*. To our knowledge, this is the first report of a prospective EWAS of CVD in a type 1 diabetes cohort. Our results support our hypothesis that DNAm-CVD associations differ by glycemic exposure. Furthermore, adjustment for established cardiometabolic risk factors led to less attenuation of DNAm-CVD associations in participants with higher HbA1c compared to lower HbA1c, suggesting epigenetic regulation may make a greater independent contribution to CVD pathogenesis under conditions of higher glycemic exposure.

Hypermethylation of cg21823999 in *GPM6A* and hypomethylation of cg23621817 in *CHRNA9,* both loci involved in calcium channel activity, were associated with increased CVD risk only in participants with higher HbA1c at baseline. Calcium channel activity is responsible for vascular smooth muscle tone and reactivity via control of vasoconstriction and vasorelaxation [17]. While diabetes has been linked to aberrant calcium signaling [18], *GPM6A* and *CHRNA9* have not previously been specifically implicated in contributing to CVD risk in diabetes. However, there is evidence *GPM6A* and *CHRNA9* may be involved in myocardial development and repair [19, 20] and *GPM6A* is differentially expressed with respect to acute myocardial infarction [21]. Thus, it is plausible that glycemic exposure modifies epigenetic regulation of *GPM6A* and *CHRNA9* expression to affect downstream CVD risk. Experimental studies on the impact of altered glucose state on *GPM6A* and *CHRNA9* expression are needed to test that hypothesis.

We identified cis-meQTLs significantly associated with DNAm at three of the CVD-associated CpGs and validated those meQTLs using the GoDMC database. There were significant meQTLs for cg14524754 in *B3GNTL1* which, while not well-characterized, has been linked to immunological changes related to lung and colorectal cancers [22], and in *TBCD*, which encodes tubulin-folding cofactor D, a chaperone protein necessary for β-tubulin folding [23]. In a heterodimer with α-tubulin, β-tubulin forms microtubules which not only play a key role in vascular remodeling under pathological conditions but also regulate inflammation [24]. Our findings support further investigation into the role of *B3GNTL1* and *TBCD* in CVD pathogenesis in type 1 diabetes. We also identified significant meQTLs for cg07147033 in *MIB2* and *FNDC10. MIB2* is involved in Notch signaling, promoting cell proliferation and activation, protection against cytokine-induced cell death [25], and is in the lipid and atherosclerosis KEGG pathway [15]. In the current study, in participants with HbA1c < 8.9%, DNAm at cg07147033 in *MIB2* was strongly associated with worse levels of several cardiometabolic risk factors, including BMI, eGDR, non-HDL cholesterol, triglycerides, AER, and eGFR. Additionally, much of the association between cg07147033 methylation and CVD risk was explained by adjustment for those risk factors, particularly non-HDL cholesterol.

In contrast, our results suggest DNAm may make a greater independent contribution to CVD risk under conditions of higher glycemic exposure. In those with HbA1c ≥ 8.9%, in addition to DNAm of *GPM6A* and *CHRNA9* already described above, there were also associations between DNAm and CVD in loci involved in cellular response to stress (*GAS7*), protein localization (*GET4*), and cell cycle (*MAD1L1*) [16]. The direction of DNAm-CVD associations also differed by HbA1c. In those with lower HbA1c, for all top CpGs, hypermethylation was associated with increased CVD risk, while in those with higher HbA1c, we observed both hypo- and hypermethylation of CpGs associated with increased CVD risk. Prior studies have suggested that hypermethylation may be more characteristic of atherosclerosis, while hypomethylation may be more closely related to aging processes [26]. While more research is needed to support those hypotheses, our results could mean that epigenetically regulated accelerated aging pathways contribute to CVD risk under conditions of higher glycemic exposure. Indeed, accelerated aging related to hyperglycemia has previously been proposed as a potential explanation for the increased risk of vascular disease in type 1 diabetes [27].

We [7] and others [6] have previously shown DNAm is associated with glycemic control in type 1 diabetes. In DCCT/EDIC, prior mean HbA1c was associated with DNAm at several CpGs and methylation at those sites was also prospectively associated with microvascular complications [6]. Given the differences in study designs between EDC and DCCT/EDIC, we are not able to perform similar analyses using prior mean HbA1c but have instead adjusted for concurrent HbA1c. Nonetheless, the results of the current analyses are consistent with our prior data showing differences in clinical risk factors for CVD by level of glycemic exposure [13] and suggest that epigenetic regulation of factors related to CVD pathogenesis may differ by glycemic exposure as well. A more comprehensive examination of the role of glycemia in the association between methylation and CVD is warranted and will be the subject of future analyses in the EDC cohort.

Our results overlap somewhat with prior large EWAS in general population studies. The Cohorts for Heart and Aging Genetic Epidemiology (CHARGE) Consortium has performed the largest prospective EWAS of coronary heart disease to date and reported associations with DNAm in *PTPRN2* and *MAD1L1* [8]. Multi-Ethnic Study of Atherosclerosis (MESA) similarly demonstrated *PTPRN2* expression is associated with coronary artery calcification and carotid plaque score [28]. Our observation that methylation of cg02768721 in *PTPRN2* has a suggestive association with CVD only in EDC participants with lower HbA1c corresponds to those associations between methylation of *PTPRN2* and CVD in the general population, which has low glycemic exposure on average.

In addition to findings discussed above, we identified associations between DNAm and CVD at several CpGs that were driven by a small number of participants with extreme methylation probe *β* values (data shown in the Additional file 1). Upon examination of their clinical characteristics, all had either HbA1c > 9% or advanced kidney disease at baseline and three of the eight individuals had a greater proportion of extreme probe *β* values than the overall cohort. It has been shown that methylation outlier burden likely reflects biological age and is unlikely to be a result of technical artifacts [29]. Thus, the biological plausibility of the corresponding loci, including *FBXO27*, which is involved in autophagy [30] and highly expressed in cardiomyocytes [31]; *CARD9,* which encodes a protein that regulates cell apoptosis and NF-kappaB activation [32]; and *NLN,* which is involved in the renin–angiotensin–aldosterone system [33], suggest they may warrant further study in external cohorts.

### Strengths and limitations

Our study has several strengths, including the use of data from a well-characterized, exclusively type 1 diabetes cohort with long-term follow-up to ascertain CVD incidence. The EDC cohort is epidemiologically representative of the childhood-onset type 1 diabetes population of Allegheny County, Pennsylvania [34]. The prospective study design avoids the possibility of reverse causation. Another strength is that CVD events were verified using death certificates and medical records by physician reviewers masked to risk factor status. A further strength is the availability of traditional cardiometabolic risk factor data, facilitating examination of cardiometabolic phenotypes for the CVD-associated DNAm. We also assessed DNAm-CVD associations separately by level of glycemic exposure, an important potential effect modifier in type 1 diabetes.

Limitations of our study include use of whole blood for methylation measurement and a lack of tissue-specific data. However, DNAm in whole blood is commonly examined in epidemiologic studies such as ours, due to ease of specimen collection and because it facilitates detection of multiple physiologic pathways that lead to complex phenotypes like CVD. We used a standard method to account for blood cell type composition in our analyses. Another limitation is the lack of long-term glycemic exposure data prior to DNA collection, so we are unable to assess the temporal association between glycemic exposure and differential methylation. The sample size of our study is relatively small with limited power for the secondary outcomes MACE and major CAD in particular, restricting our ability to identify strong associations. Thus the results should be validated in other cohorts as data become available. Additionally, due to the small sample size, we applied a more liberal cut-off of FDR ≤ 0.20 for including CpGs in the post hoc functional analyses to facilitate our goal of identifying potentially important pathways. This approach is similar to other prior epidemiological studies of DNAm which have used the approach of applying a more liberal FDR cut point for inclusion of CpGs in enrichment analyses [35, 36]. Finally, the EDC cohort is 98% white/European ancestry, primarily due to the demographics of Allegheny County, Pennsylvania, USA (< 15% black/African American) and historically lower incidence of type 1 diabetes among black individuals [37], so our results may not apply to more diverse populations.

## Conclusions

We present novel evidence that DNAm at several CpGs may be associated with future CVD risk in type 1 diabetes and contribute to risk independently of traditional cardiometabolic risk factors. Importantly, our findings suggest epigenetic modification of *GPM6A* and *CHRNA9*, both involved in calcium channel activity and development, may play a role in the pathophysiology of CVD under conditions of higher glycemic exposure in type 1 diabetes. While experimental data and validation in external populations are needed, our observation of such glycemia-dependent associations supports the existence of heterogeneous pathways to CVD development in type 1 diabetes and may provide insight into new targets for intervention to prevent or delay CVD in this high-risk population.

## Methods

### Study population

The Pittsburgh Epidemiology of Diabetes Complications (EDC) study is a prospective cohort study of childhood-onset (< 17 years old) T1D. All participants in the parent cohort (*n* = 658) were diagnosed with T1D, or seen within one year of diagnosis, at Children’s Hospital of Pittsburgh between 1950 and 1980. The cohort has been described in detail [38] and a study timeline is shown in Fig. 1. Participants have been followed since 1986–1988, with biennial examinations and questionnaires for the first ten years and thereafter with biennial questionnaires and further examinations in 2004–2006, 2011–2013, and 2016–2018. DNA was collected at study visits between 1988 and 1998: 86% of the DNA specimens were collected at the 1988–1990 visit, 9% at the 1990–1992 visit, and the remaining 5% between 1992 and 1998. The date of each participant’s DNA specimen defines their baseline for these analyses.

A diagram detailing derivation of the final analytic sample is in Fig. 1. Of 496 participants consenting to blood leukocyte DNA collection, 436 European ancestry participants had suitable quality DNA available for methylation arrays remaining in 2020. After excluding participants not passing methylation quality control (*n* = 11), one randomly selected individual from each of 10 first-degree relative pairs, two outliers from genetic principal components analysis, and one randomly selected individual from each of 2 technical duplicate specimen pairs the final sample comprised 411 participants.

### DNA methylation arrays, quality control, and data processing

High molecular weight DNA was isolated from whole blood-derived leukocytes. DNAm was assayed using Illumina Infinium MethylationEPIC BeadChip arrays (Illumina, San Diego, CA, USA) [39] at the University of Virginia Center for Public Health Genomics, Genomic Sciences Laboratory using the standard Illumina methylation array protocol. Quality control methods were implemented in two packages in R (v4.1.0; R Core Team 2021), *minfi* v1.32.0 [40] and *SeSAMe* v1.8.10 [41]. The pipeline, including the specific functions used, is summarized in Additional file 1: Fig. S1. The EPIC output Intensity Data (IDAT) file contained raw probe intensity data, as well as the control probe information needed for quality control. Using *minfi*, the IDAT files were loaded and converted into an RGSet object and thence to a GMSet object based on the Illumina 1.0 B5 hg38 annotation release. The minfiQC function was used to check for outlier samples of potentially poorer quality based on: (1) median intensity of methylated (M) versus unmethylated (U) probes (minfiQC() function, outlier cut-off = 3 stdev); (2) median X, Y chromosome probe signals for karyotype prediction (getSex() function cut-off = − 2); (3) sample probe detection rate (detectionP() function, *p* value < 0.01). The third step was repeated after dropping 72,868 low quality probes (probe detection rate < 95% as described below) and a previously published curated list of 95,923 probes recommended for exclusion as detailed by Zhou et al. [42]. Specifically, the list curated by Zhou et al. excludes probes with: inconsistent mapping; SNPs in the extension base that cause a color channel switch from the official annotation; non-unique 30bp3’ subsequence; 5bp3’ subsequence overlap with any of the SNPs with global minor allele frequency > 1%; and extension base inconsistent with the specified color channel or CpG.

To confirm and expand the *minfi* QC, a second pipeline of QC checks used functions in the *SeSAMe* package [41]. First, the RGset object was converted to a SigSet object using the RGSetChannelSetToSigSet() function. Then, the pipeline checked: (1) mean sample probe intensity distribution by sample, mean(M + U) (sesameQC() function); (2) sample bisulfite GCT score for completeness of bisulfite conversion (sesameQC() function); (3) inferred sex karyotype based on chromosome X and Y probes (inferredSexKaryotypes() function); (4) detection of duplicate samples using SNP genotype data extracted from a subset of the probes. Finally, probe quality checks were performed based on individual probe detection p values. In this check, any probe that was not detected in at least 95% of samples was dropped (72,868 unique probes excluded). Cryptic sample duplicates were inferred using KING [43] applied to the SNP genotype data extracted from the Illumina ‘spiked-in’ SNP probes (*n* = 44) and additional probes that manifest a reproducible pattern of genotype-stratified methylation signals driven by a flanking SNP (*n* = 865 SNPs in total). After the QC described above and further restricting analysis to probes mapped to autosomal chromosomes, 683,597 of the original 865,918 probes on the EPIC array were analyzed.

The final methylation fraction β values for analysis were generated using the following pipeline steps in *SeSAMe* [41]: (1) background subtraction based on normal-exponential deconvolution using out-of-band probes (noob); (2) dye bias correction in the two-color channels through nonlinear scaling (dyeBiasCorrTypeINorm); (3) probe quality masking based on the curated exclusion list (qualityMask); (4) detection masking at individual probe/sample level using pooBAH p value out-of-band array hybridization (detectionMask); (5) beta calculation, M/M + U (getBetas); (6) drop samples and probes identified during quality control pre-processing. Finally, for each probe we excluded *β* values >  ± 3 standard deviations from the mean to remove extreme outliers for the primary analysis. As DNA was isolated from whole blood-derived leukocytes, variable cell type composition and differential methylation states in a sample could confound association tests. Thus, cell type compositions variables were estimated using the estimateCellCounts2 function from the R package *FlowSorted.Blood.EPIC* [44], using an IDOL-optimized probe set [45] derived from the original Houseman method [46] which has been shown to be an optimal algorithm for cell-type deconvolution in EWAS using whole blood specimens [47].

### Ascertainment of cardiovascular outcomes

Follow-up time for each participant was calculated from the study visit during which their DNA was isolated (baseline) until CVD incidence or censoring (31 December 2018 or last follow-up). CVD was defined as the first instance of CVD death, nonfatal myocardial infarction (MI, including clinical events and subclinical myocardial infarction on ECG, i.e., Minnesota code 1.1 or 1.2), nonfatal stroke, coronary revascularization procedure, blockage ≥ 50%, ischemic EGC at EDC study visit (Minnesota codes 1.3, 4.1–4.3, 5.1–5.3, 7.1), or EDC physician-diagnosed angina. Two secondary outcomes were also examined: major adverse cardiovascular events (MACE, first instance of CVD death, nonfatal MI or nonfatal stroke) and major coronary artery disease (CAD, first instance of CAD death or nonfatal MI). Fatal events were ascertained using medical records, death certificates, autopsy reports, and/or interview with next of kin and classified according to the Diabetes Epidemiology Research International (DERI) system [48]. Nonfatal events were confirmed with medical records.

### CVD EWAS

Participants with prevalent total CVD (*n* = 43), MACE (*n* = 14), or major CAD (*n* = 11) at the time of DNA collection were excluded from each respective analysis. We performed a time-to-event EWAS for total CVD incidence with CpGs on chromosomes 1–22 using Cox regression. Each CpG probe β-value was modeled as the main independent variable and adjusted for type 1 diabetes duration, sex, pack-years of smoking, cell type composition variables, plate/run number, GCT bisulfite score, DNA extraction method, and well position. As one of our aims was to identify genetic variants associated with DNAm, we also included the first two ancestry principal components based on GWAS data [49] as covariates in the EWAS. EDC is an exclusively childhood-onset (< 17 years) type 1 diabetes cohort; thus, age and diabetes duration are highly correlated (*r* = 0.86, *p* < 0.0001). As type 1 diabetes duration is the exposure of greater interest in the current analysis, the results we present were adjusted for diabetes duration only. However, results remained the same in alternative models adjusting for age instead of diabetes duration. Separate EWAS of the secondary outcomes MACE and major CAD were also performed using the same methods described above. As DNAm outliers could be associated with extreme phenotypes, we also performed alternative exploratory analyses including extreme probe *β* values (> ± 3 standard deviations from the mean). Given the sample size of the cohort, potentially important associations with small effect sizes would be disregarded if we only applied a conservative statistical significance cut-off. Thus, a Benjamini–Hochberg false discovery rate (FDR) ≤ 0.05 was considered significant and FDR ≤ 0.10 was considered nominally significant. Additionally, to avoid overlooking potentially important functional relationships, a more liberal FDR ≤ 0.20 was considered suggestive and warranting inclusion in the secondary post hoc functional analyses. As the traditional genomic inflation factor λ has been shown to overestimate test statistic inflation in EWAS [50], we used the *bacon* method (λ.*bacon*), a Bayesian method developed by Iterson et al*.* specifically for EWAS [50], to estimate inflation if traditional *λ* > 1.10.

### HbA1c-stratified EWAS and cardiometabolic risk factor analyses

We repeated the EWAS for total CVD stratified by HbA1c to determine whether CVD-associated DNAm differed by level of glycemic control. Because there is no known optimal HbA1c cut point at which the CVD-DNAm association is modified, median baseline HbA1c (8.9% [74 mmol/mol]) was used as the cut point for stratification, ensuring approximately equal sample size in each stratum. We then assessed associations between DNAm at CpGs considered at least nominally suggestive (FDR ≤ 0.10) and levels of baseline cardiometabolic risk factors using linear regression separately by median HbA1c (8.9%). Cardiometabolic risk factors analyzed were HbA1c, body mass index (BMI), estimated glucose disposal rate (eGDR), HDL cholesterol, non-HDL cholesterol, triglycerides, systolic and diastolic blood pressures (SBP and DBP), pulse rate, albumin excretion rate (AER), and estimated glomerular filtration rate (eGFR). Details regarding ascertainment of cardiometabolic risk factors have been published previously [51] and are described in the Additional file 1. In separate minimally adjusted linear models, each cardiometabolic risk factor was the dependent variable and the methylation *β* value at each CVD-associated CpG was the independent variable, adjusting for type 1 diabetes duration, sex, pack-years of smoking, and cell type composition variables. An adjusted *p* value ≤ 0.0005 (0.05/99 comparisons, i.e., 9 CpGs × 11 risk factors) was considered conservatively statistically significant, while *p* ≤ 0.05 was nominally significant. Finally, for each CpG assessed, we re-fit Cox models for time-to-CVD, adjusting for cardiometabolic risk factors, selected using backward selection, to obtain cardiometabolic risk factor-independent estimates of the DNAm-CVD associations. Risk factors were entered into the models if they were univariately associated with the CpG at *p* ≤ 0.10 and were retained in the Cox model if *p* ≤ 0.05.

### Post hoc functional analyses

For CpG associated with CVD at FDR ≤ 0.20 in EWAS, we identified methylation quantitative trait loci (meQTLs) via GWAS using existing imputed genotyping array data in the EDC cohort [49]. Genotyping was performed in *n* = 453 EDC participants using the Infinium HumanCore Exome-24 BeadChip (Illumina, San Diego, CA, USA), following the manufacturer’s protocol. After quality control and exclusions, quality genotyping data were available for *n* = 422. Determination of the analytic sample has been described previously [52]. Minimac3/ Minimac3-omp version 1.0.14 was used for imputation with the 1000 Genomes (1KG) Phase 3 version 5 reference panel (updated Oct 20, 2015). In the *n* = 411 EDC participants included in the DNAm analysis, we performed GWAS of the CVD-associated CpGs with FDR ≤ 0.20. DNAm probe β-values were modeled as the dependent variable and SNP dosage (additive coding) as independent variables in separate linear models, adjusting for sex, T1D duration, pack-years of smoking, estimated cell type composition, and the first three ancestry principal components. Genome-wide significant SNPs at *p* ≤ 5 × 10^–8^ were identified as meQTLs for the corresponding CpG. Results were compared to the Genetics of DNA Methylation Consortium (GoDMC) database of meQTLs [53] for external validation. Finally, we looked for evidence of the meQTLs’ effects on gene expression by determining whether the meQTLs were annotated as whole blood eQTLs in the Genotype-Tissue Expression (GTEx) Project database using a significant threshold of *p* < 0.0005 (*p* = 0.05/93 meQTLs assessed). The data used for the analyses described here were obtained from the GTEx Portal on 6 September 2022.

Gene information was obtained from Illumina annotation (March 2020, reference genome GRCh38/hg38). We performed gene set enrichment analysis (GSEA) using the GOmeth function in the *missMethyl* package for R with the *GO.db* and *KEGG.db* annotation packages [54] to identify enrichment of terms associated with ≥ 2 loci with CVD-associated DNAm. For all loci associated with CpGs with FDR ≤ 0.20, we identified a Reactome Functional Interaction (FI) network [16] using Cytoscape [55]. Modules within the network were identified using a spectral algorithm for clustering [56]; we performed Reactome pathway analysis on the resulting modules with ≥ 3 total nodes including ≥ 2 candidate loci.

## Supplementary Information

**Additional file 1. Supplementary Methods:** Cardiometabolic risk factors. **Supplementary Results:** Extreme probe β values. **Table S1.** All CpGs with DNA methylation associated with time to major adverse cardiovascular event (MACE) and major coronary artery disease (CAD) at FDR <0.20 included in post hoc functional analyses. **Table S2.** Top 5 CpGs with DNA methylation associated with time to total CVD, MACE, and major CAD incidence in the EDC, including extreme probe β outliers. **Table S3.** Cardiometabolic risk factors and methylation at CVD associated differentially methylated positions (FDR ≤ 0.10) – HbA1c <8.9%. **Table S4.** Cardiometabolic risk factors and methylation at CVD-associated differentially methylated positions (FDR ≤ 0.10) – HbA1c ≥8.9%. **Table S5.** Associations between DNA methylation and total CVD after adjustment for traditional cardiometabolic risk factors by median HbA1c at baseline. **Table S6.** Genome-wide significant (p<5.0E-8) methylation quantitative trait loci (meQTLs) for CVD-associated methylation identified in the EDC cohort. **Table S7.** Gene ontology (GO) terms with p<0.05 for total CVD in EDC participants with HbA1c ≥8.9%. **Table S8.** Loci included in Reactome Functional Interaction network analysis (alphabetical). **Table S9.** Significantly enriched Reactome pathways identified in functional interaction network analysis of loci containing CpGs with DNA methylation associated with CVD incidence at FDR<0.20. **Figure S1.** DNA methylation quality control (QC) pipeline used in the EDC study. **Figure S2.** Kaplan-Meier curves of proportion free of each CVD outcome. **Figure S3.** Manhattan plot (A) for the epigenome-wide association of DNAm with time-to-CVD event after excluding probe beta outliers and gene track plots for CpGs with FDR<0.20 (B-D) in the EDC study. **Figure S4.** QQ plot for the epigenome-wide association of DNAme with time-to-CVD event. **Figure S5.** QQ plot for the epigenome-wide association of DNAme with time-to-CVD event, all observations included.

## Data Availability

Data, code, and associated documentation are available from the corresponding author on reasonable request. In accordance with University of Pittsburgh and Institutional Review Board policies and regulations, a data-sharing agreement is required.
