# Supplementary material for: DNA methylation and 28-year cardiovascular disease risk in type 1 diabetes: the Epidemiology of Diabetes Complications (EDC) cohort study
Source: Clin Epigenetics. 2023 Aug 2;15:122. doi: 10.1186/s13148-023-01539-0 (PMC10394855; doi:10.1186/s13148-023-01539-0)
Supplement: Supplementary file 1 — Additional file 1. Supplementary Methods: Cardiometabolic risk factors. Supplementary Results: Extreme probe β values. Table S1. All CpGs with DNA methylation associated with time to major adverse cardiovascular event (MACE) and major coronary artery disease (CAD) at FDR <0.20 included in post hoc functional analyses. Table S2. Top 5 CpGs with DNA methylation associated with time to total CVD, MACE, and major CAD incidence in the EDC, including extreme probe β outliers. Table S3. Cardiometabolic risk factors and methylation at CVD associated differentially methylated positions (FDR ≤ 0.10) – HbA1c <8.9%. Table S4. Cardiometabolic risk factors and methylation at CVD-associated differentially methylated positions (FDR ≤ 0.10) – HbA1c ≥8.9%. Table S5. Associations between DNA methylation and total CVD after adjustment for traditional cardiometabolic risk factors by median HbA1c at baseline. Table S6. Genome-wide significant (p<5.0E-8) methylation quantitative trait loci (meQTLs) for CVD-associated methylation identified in the EDC cohort. Table S7. Gene ontology (GO) terms with p<0.05 for total CVD in EDC participants with HbA1c ≥8.9%. Table S8. Loci included in Reactome Functional Interaction network analysis (alphabetical). Table S9. Significantly enriched Reactome pathways identified in functional interaction network analysis of loci containing CpGs with DNA methylation associated with CVD incidence at FDR<0.20. Figure S1. DNA methylation quality control (QC) pipeline used in the EDC study. Figure S2. Kaplan-Meier curves of proportion free of each CVD outcome. Figure S3. Manhattan plot (A) for the epigenome-wide association of DNAm with time-to-CVD event after excluding probe beta outliers and gene track plots for CpGs with FDR<0.20 (B-D) in the EDC study. Figure S4. QQ plot for the epigenome-wide association of DNAme with time-to-CVD event. Figure S5. QQ plot for the epigenome-wide association of DNAme with time-to-CVD event, all observations included. [file 13148_2023_1539_MOESM1_ESM.docx]

**Additional file 1**

**DNA Methylation and 28-Year Cardiovascular Disease Risk in Type 1 Diabetes: The Epidemiology of Diabetes Complications (EDC) Study**

Rachel G. Miller, Josyf C. Mychaleckyj, Suna Onengut-Gumuscu, Eleanor Feingold, Trevor J. Orchard, Tina Costacou

**Supplementary Methods: Cardiometabolic risk factors**

We assessed associations between CVD-associated CpGs with FDR≤0.10 from the total CVD EWAS and continuous baseline (i.e., study visit of DNA collection) cardiometabolic risk factors using linear regression. The cardiometabolic risk factors assessed were HbA1c, body mass index (BMI), estimated glucose disposal rate (eGDR), HDL cholesterol, non-HDL cholesterol, triglycerides, systolic and diastolic blood pressures (SBP and DBP), pulse rate, albumin excretion rate (AER), and estimated glomerular filtration rate (eGFR). Details regarding ascertainment of cardiometabolic risk factors have been published previously (1). Fasting blood samples were obtained to measure HbA1, lipids, and serum creatinine. HbA1 values were converted to DCCT-aligned values using a regression equation derived from duplicate assays (DCCT HbA1c=0.14 + 0.83[EDC HbA1]) (2). Total cholesterol and triglycerides were determined enzymatically (3,4). HDL cholesterol was determined using a modified precipitation technique (5). Non-HDL cholesterol was calculated by subtracting HDL cholesterol from total cholesterol. Blood pressure was measured according to the Hypertension Detection and Follow-Up protocol with a random-zero sphygmomanometer (6). Pulse rate (beats/minute) was determined by palpitating the radial pulse for 30 seconds and multiplying by two. Urinary albumin was measured by immunonephelometry (7). Albumin excretion rate (AER) was calculated for each of three timed urine samples (24-hour, overnight, and 4-hour collections obtained over a two-week period); the median of the three AERs was used in analyses. Serum creatinine was measured using an Ectachem 400 Analyzer (Eastman Kodak Co.) and glomerular filtration rate was estimated by the CKD-EPI creatinine equation (8). Height and weight were measured using standard methods to calculate BMI. Smoking history, insulin regimen, lipid-lowering and blood pressure-lowering medication use were self-reported via questionnaire. Insulin dose was calculated as total insulin units per day divided by body weight (kg).

**Supplementary Methods References:**

1. Miller RG, Orchard TJ, Costacou T. Risk factors differ by first manifestation of cardiovascular disease in type 1 diabetes. Diabetes Res Clin Pract. 2020;163:108141.

2. Prince CT, Becker DJ, Costacou T, Miller RG, Orchard TJ. Changes in glycaemic control and risk of coronary artery disease in type 1 diabetes mellitus: findings from the Pittsburgh Epidemiology of Diabetes Complications Study (EDC). Diabetologia. 2007 Nov;50(11):2280–8.

3. Allain CC, Poon LS, Chan CSG, Richmond W, Fu PC. Enzymatic determination of total serum cholesterol. Lipids. 1974;20(4):470–5.

4. Bucolo G, David H. Quantitative determination of serum triglycerides by the use of enzymes. Clin Chem. 1973;19(5):476–82.

5. Warnick GR, Albers JJ. Heparin--Mn2+ quantitation of high-density-lipoprotein cholesterol: an ultrafiltration procedure for lipemic samples. Clin Chem. 1978;24(6):900–4.

6. The hypertension detection and follow-up program. Hypertension detection and follow-up program cooperative group. Prev Med. 1976;5:207–15.

7. Ellis D, Coonrod BA, Dorman JS, Kelsey SF, Becker DJ, Avner ED, et al. Choice of urine sample predictive of microalbuminuria in patients with insulin-dependent diabetes mellitus. Am J Kidney Dis. 1989;13(4):321–8.

8. Levey AS, Stevens LA, Schmid CH, Zhang YL, Castro AF, Feldman HI, et al. A new equation to estimate glomerular filtration rate. Ann Intern Med. 2009;150(9):604–12.

**Supplementary Results: Extreme probe β values**

As DNAm outliers could be associated with extreme phenotypes, we also performed an alternative exploratory analysis including extreme probe β values. In the alternative analysis, higher levels of DNAm at two CpGs (cg06899985, *IGDCC4,* p=3.65x10^-8^ and cg14730085, *FBXO27,* p=7.84x10^-8^) was significantly associated with increased total CVD incidence (Supplementary Table 2). The corresponding QQ plot is in Supplementary Figure 5 (λ =1.047). There were four individuals with extreme values for cg06899985 and six individuals for cg14730085, two of which overlapped. Thus, a total of eight participants had extreme values for either CpG. All eight participants developed CVD during follow-up and most had prevalent kidney disease and HbA1c >9% at baseline but did not have any remarkable shared characteristics in batch covariates. However, three of the eight participants had a larger proportion of probe β values > ± 3 SD from the mean compared to the overall proportion (10-16% versus 1.3%, respectively). When the eight individuals were excluded, associations between cg06899985 and cg14730085 and CVD were markedly attenuated (p=0.50 and 0.01, respectively).

**Supplementary Table 1. All CpGs with DNA methylation associated with time to major adverse cardiovascular event (MACE) and major coronary artery disease (CAD) at FDR <0.20 included in *post hoc* functional analyses**

| Outcome | ID | Chr | Location | CpG Position (hg38) | Gene Symbol | Location Relative to Gene | β per 5% methylation | SE | p-value | FDR |
| --- | --- | --- | --- | --- | --- | --- | --- | --- | --- | --- |
| MACE | cg26234602 | 17 | OpenSea | 5005472 | *KIF1C* | Body | 3.127 | 0.606 | 2.49E-07 | 0.14 |
|  | cg16278436 | 1 | OpenSea | 17167886 | *(intergenic)* | n/a | 0.701 | 0.139 | 4.58E-07 | 0.14 |
|  | cg05502662 | 15 | Island | 60479117 | *NARG2* | 5’UTR; 1^st^Exon | 10.037 | 2.012 | 6.11E-07 | 0.14 |
|  | cg24048949 | 19 | S_Shelf | 2893181 | *(intergenic)* | n/a | 0.607 | 0.124 | 1.02E-06 | 0.17 |
|  | cg05193189 | 12 | OpenSea | 132664950 | *POLE* | Body | 0.633 | 0.132 | 1.64E-06 | 0.19 |
|  | cg00770443 | 12 | OpenSea | 108218067 | *WSCD2* | Body | 0.649 | 0.137 | 2.31E-06 | 0.19 |
|  | cg00621805 | 7 | OpenSea | 74566884 | *GTF2IRD1* | Body | -0.771 | 0.163 | 2.32E-06 | 0.19 |
|  | cg24686957 | 6 | OpenSea | 5214716 | *LYRM4* | Body | 0.920 | 0.195 | 2.45E-06 | 0.19 |
|  | cg15409399 | 5 | Island | 72956662 | *FCHO2* | Body | -1.666 | 0.354 | 2.55E-06 | 0.19 |
| Major CAD | cg24048949 | 19 | S_Shelf | 2893181 | (intergenic) | n/a | 0.663 | 0.131 | 4.27E-07 | 0.11 |
|  | cg15409399 | 5 | Island | 72956662 | *FCHO2* | Body | -1.914 | 0.381 | 5.14E-07 | 0.11 |
|  | cg11188031 | 5 | OpenSea | 157058636 | *HAVCR1* | TSS200 | 4.896 | 0.981 | 5.97E-07 | 0.11 |
|  | cg00621805 | 7 | OpenSea | 74566884 | *GTF2IRD1* | Body | -0.854 | 0.173 | 7.37E-07 | 0.11 |
|  | cg26234602 | 17 | OpenSea | 5005472 | *KIF1C* | Body | 3.206 | 0.649 | 7.79E-07 | 0.11 |
|  | cg24686957 | 6 | OpenSea | 5214716 | *LYRM4* | Body | 1.021 | 0.208 | 9.48E-07 | 0.11 |
|  | cg26154714 | 1 | N_Shore | 26992126 | (intergenic) | n/a | 0.725 | 0.150 | 1.26E-06 | 0.12 |
|  | cg11047111 | 1 | Island | 10430286 | *APITD1* | 5'UTR; 1stExon; TSS1500 | -2.483 | 0.526 | 2.35E-06 | 0.19 |
|  | cg09537606 | 5 | OpenSea | 157978265 | (intergenic) | n/a | 1.302 | 0.277 | 2.50E-06 | 0.19 |
|  | cg19046189 | 19 | S_Shore | 48813474 | *HSD17B14* | Body | 1.076 | 0.232 | 3.44E-06 | 0.19 |
|  | cg11143292 | 17 | OpenSea | 7642732 | (intergenic) | n/a | 0.644 | 0.140 | 4.19E-06 | 0.19 |
|  | cg03585572 | 15 | OpenSea | 77769857 | *LINGO1* | 5'UTR | 0.751 | 0.163 | 4.22E-06 | 0.19 |
|  | cg06653878 | 9 | S_Shore | 95318335 | *FANCC* | TSS1500 | -3.081 | 0.673 | 4.63E-06 | 0.19 |
|  | cg17848458 | 1 | S_Shore | 9200689 | (intergenic) | n/a | 0.711 | 0.156 | 5.06E-06 | 0.19 |
|  | cg00617180 | 6 | OpenSea | 44674313 | (intergenic) | n/a | 0.536 | 0.118 | 5.16E-06 | 0.19 |
|  | cg08556156 | 10 | OpenSea | 103507640 | *NEURL1; NEURL1-AS1* | Body; Body | 0.731 | 0.161 | 5.37E-06 | 0.19 |
|  | cg00770443 | 12 | OpenSea | 108218067 | *WSCD2* | Body | 0.657 | 0.145 | 5.57E-06 | 0.19 |
|  | cg21611056 | 20 | N_Shore | 3795524 | *CDC25B* | TSS1500; Body | -0.965 | 0.213 | 5.64E-06 | 0.19 |
|  | cg05502662 | 15 | Island | 60479117 | *NARG2* | 5'UTR; 1stExon | 9.939 | 2.190 | 5.67E-06 | 0.19 |
|  | cg05193189 | 12 | OpenSea | 132664950 | *POLE* | Body | 0.635 | 0.140 | 5.71E-06 | 0.19 |
|  | cg11713800 | 6 | Island | 41653212 | *MDFI* | Body | 1.138 | 0.251 | 5.72E-06 | 0.19 |

**Supplementary Table 2.** Top 5 CpGs with DNA methylation associated with time to total CVD, MACE, and major CAD incidence in the EDC, including extreme probe β outliers

| Outcome | ID | Chr | Location (hg38) | CpG Position | Gene Symbol | Location Relative to Gene | β per 5% methylation | SE | p-value | False Discovery Rate |
| --- | --- | --- | --- | --- | --- | --- | --- | --- | --- | --- |
| Total CVD | cg06899985 | 15 | 65396959 | Island | *IGDCC4* | Body | 0.740 | 0.134 | 3.65E-08 | 0.01 |
|  | cg14730085 | 19 | 39031907 | Island | *FBXO27* | Body | 1.879 | 0.350 | 7.84E-08 | 0.01 |
|  | cg19759282 | 2 | 174486920 | Island | *GPR155* | 5'UTR; 1stExon | 3.426 | 0.639 | 8.14E-08 | 0.01 |
|  | cg12779717 | 1 | 117569066 | OpenSea | n/a | Intergenic | -0.920 | 0.172 | 8.29E-08 | 0.01 |
|  | cg18815943 | 1 | 47416641 | Island | *FOXE3* | 1stExon | 0.724 | 0.135 | 8.35E-08 | 0.01 |
|  |  |  |  |  |  |  |  |  |  |  |
| MACE | cg06125996 | 5 | 65818753 | OpenSea | *NLN* | Body | -1.010 | 0.165 | 9.06E-10 | 0.001 |
|  | cg03552151 | 20 | 34515778 | N_Shore | *DYNLRB1* | TSS1500 | 2.092 | 0.350 | 2.30E-09 | 0.001 |
|  | cg01410279 | 1 | 171652800 | OpenSea | *MYOC* | TSS200 | -0.500 | 0.086 | 7.44E-09 | 0.002 |
|  | cg01835926 | 17 | 18951416 | OpenSea | *SLC5A10* | TSS1500; 5'UTR | -1.148 | 0.210 | 4.73E-08 | 0.01 |
|  | cg02460371 | 1 | 150294183 | Island | *MRPS21* | 5'UTR; 1stExon | 1.572 | 0.294 | 9.30E-08 | 0.01 |
|  |  |  |  |  |  |  |  |  |  |  |
| Major CAD | cg06125996 | 5 | 65818753 | OpenSea | *NLN* | Body | -1.100 | 0.169 | 7.58E-11 | 0.0001 |
|  | cg01835926 | 17 | 18951416 | OpenSea | *SLC5A10* | TSS1500; 5'UTR | -1.234 | 0.216 | 1.08E-08 | 0.0037 |
|  | cg05163804 | 9 | 136368121 | S_Shore | *CARD9* | Body;Body | -0.970 | 0.173 | 2.21E-08 | 0.01 |
|  | cg02460371 | 1 | 150294183 | Island | *MRPS21* | 5'UTR; 1stExon | 1.679 | 0.309 | 5.70E-08 | 0.01 |
|  | cg11581475 | 19 | 43620571 | S_Shore | *ZNF428* | TSS1500 | -1.287 | 0.247 | 1.77E-07 | 0.02 |

**Supplementary Table 3.** Cardiometabolic risk factors and methylation at CVD-associated differentially methylated positions (FDR≤0.10) – HbA1c <8.9%

|  |  |  | β per 5% methylation (SE) | | | | | | | | | | |
| --- | --- | --- | --- | --- | --- | --- | --- | --- | --- | --- | --- | --- | --- |
| CpG | Chr | Gene | HbA1c | Body Mass Index | Estimated glucose disposal rate | HDLc | Non-HDLc | Trigly-cerides† | Systolic blood pressure | Diastolic blood pressure | Pulse Rate | Albumin excretion rate | Estimated glomerular filtration rate |
| cg07147033 | 1 | *MIB2* | 0.10 (0.06)  p=0.096 | **0.92 (0.27)**  **p=0.0008** | **-0.47 (0.11) p=0.00004** | -1.85 (0.99)  p=0.064 | **11.24 (2.81)**  **p=0.00009** | **0.19 (0.05)**  **p=0.0002** | 1.61 (1.07) p=0.134 | 1.46 (0.82) p=0.077 | 0.87 (0.95)  p=0.362 | **0.57 (0.14) p=0.0001** | **-0.47 (0.11)**  **p=0.00004** |
| cg12324048 | 3 | (intergenic) | **0.15 (0.07)**  **p=0.048** | 0.69 (0.36)  p=0.054 | **-0.31 (0.15)**  **p=0.033** | -2.16 (1.25)  p=0.086 | **13.16 (3.48)**  **p=0.0002** | 0.11 (0.06)  p=0.085 | 0.81 (1.40)  p=0.565 | 0.41 (1.09)  p=0.708 | -1.69 (1.16)  p=0.148 | 0.21 (0.19) p=0.281 | **-0.31 (0.15)**  **p=0.033** |
| cg15883830 | 1 | (intergenic) | **0.68 (0.24)**  **p=0.0058** | 1.32 (1.22)  p=0.282 | **-1.61 (0.47)**  **p=0.0008** | 3.62 (4.28)  p=0.399 | **53.67 (11.57)**  **p=0.00008** | **0.71 (0.21)**  **p=0.001** | 1.48 (4.75)  p=0.756 | 3.98 (3.69)  p=0.282 | 6.81 (3.87) p=0.080 | **1.36 (0.63)**  **p=0.033** | **-1.61 (0.47)**  **p=0.0008** |
| cg21823999 | 4 | *GPM6A* | -0.01 (0.62)  p=0.992 | -2.19 (2.99)  p=0.466 | 2.15 (1.20)  p=0.076 | -5.82 (10.56)  p=0.583 | -48.61 (29.66)  p=0.103 | -0.56 (0.49) p=0.251 | -15.75 (11.50) p=0.173 | -13.44 (8.73)  p=0.126 | 1.21 (9.83) p=0.902 | -0.48 (1.61)  p=0.765 | 2.14 (1.20)  p=0.076 |
| cg23621817 | 4 | *CHRNA9* | **1.44 (0.68)**  **p=0.036** | -2.96 (3.33) p=0.376 | -0.90 (1.37)  p=0.511 | 22.61 (11.59) p=0.053 | -5.49 (33.56)  p=0.104 | -0.38 (0.58)  p=0.512 | -12.37 (12.82)  p=0.336 | -9.73 (10.00)  p=0.332 | 6.71 (10.82) p=0.536 | -0.07 (1.75)  p=0.968 | -0.90 (1.37)  p=0.511 |
| cg10296867 | 17 | *GAS7* | -0.18 (0.12)  p=0.140 | 0.86 (0.57)  p=0.135 | -0.30 (0.24)  p=0.209 | 1.24 (2.01) p=0.540 | 1.85 (5.82) p=0.751 | -0.01 (0.10)  p=0.899 | 2.30 (2.24)  p=0.307 | 0.71 (1.74)  p=0.684 | -3.32 (1.86) p=0.075 | 0.23 (0.30) p=0.443 | -0.30 (0.24)  p=0.209 |
| cg12570712 | 19 | (intergenic) | 0.008 (0.144)  p=0.956 | -0.19 (0.69) p=0.788 | 0.23 (0.28)  p=0.425 | 2.30 (5.26) p=0.341 | -2.62 (6.97)  p=0.708 | -0.11 (0.12)  p=0.349 | 1.44 (2.69)  p=0.593 | 0.35 (2.09)  p=0.866 | -1.38 (2.24) p=0.540 | -0.58 (0.36) p=0.111 | 0.23 (0.28) p=0.425 |
| cg01028223 | 7 | *GET4* | 0.34 (0.36) p=0.346 | 0.54 (1.77)  p=0.760 | -0.94 (0.72)  p=0.192 | 6.94 (5.96) p=0.246 | **41.75 (16.90)**  **p=0.015** | **0.63 (0.29)**  **p=0.034** | 4.93 (6.82)  p=0.471 | -2.53 (5.28)  p=0.633 | 1.30 (5.70) p=0.819 | 1.21 (0.91) p=0.185 | -0.94 (0.72) p=0.192 |
| cg10462017 | 7 | *MAD1L1* | 0.31 (0.32)  p=0.340 | -1.33 (1.55) p=0.392 | 0.29 (0.64)  p=0.651 | 2.44 (5.44)  p=0.654 | -29.33 (15.38) p=0.0585 | -0.13 (0.28)  p=0.642 | -6.52 (6.06)  p=0.284 | -2.45 (4.69) p=0.602 | 3.55 (5.07)  p=0.484 | 0.06 (0.82)  p=0.945 | 0.29 (0.64) p=0.651 |

Bold font indicates p<0.05

**Supplementary Table 4.** Cardiometabolic risk factors and methylation at CVD-associated differentially methylated positions (FDR≤0.10) – HbA1c ≥8.9%

|  |  |  | β per 5% methylation (SE) | | | | | | | | | | |
| --- | --- | --- | --- | --- | --- | --- | --- | --- | --- | --- | --- | --- | --- |
| CpG | Chr | Gene | HbA1c | Body Mass Index | Estimated glucose disposal rate | HDLc | Non-HDLc | Trigly-cerides† | Systolic blood pressure | Diastolic blood pressure | Pulse Rate | Albumin excretion rate | Estimated glomerular filtration rate |
| cg07147033 | 1 | *MIB2* | 0.11 (0.09)  p=0.238 | 0.19 (0.26)  p=0.473 | -0.07 (0.14)  p=0.631 | 0.53 (0.85)  p=0.534 | 0.71 (3.22)  p=0.825 | 0.01 (0.04)  p=0.866 | 0.06 (1.24) p=0.962 | 0.96 (0.84)  p=0.259 | -0.24 (0.77)  p=0.752 | -0.11 (0.17) p=0.499 | -0.066 (0.137) p=0.631 |
| cg12324048 | 3 | (intergenic) | 0.09 (0.11)  p=0.3995 | -0.02 (0.31)  p=0.949 | **-0.32 (0.16)**  **p=0.047** | -1.51 (1.00)  p=0.133 | **8.48 (3.69) p=0.023** | **0.13 (0.05)**  **p=0.016** | 2.37 (1.48) p=0.110 | 1.24 (1.01)  p=0.220 | 1.66 (0.92)  p=0.072 | 0.12 (0.19) p=0.545 | **-0.322 (0.161)**  **p=0.047** |
| cg15883830 | 1 | (intergenic) | 0.33 (0.35)  p=0.343 | 0.07 (1.02)  p=0.947 | -0.35 (0.54)  p=0.512 | -3.24 (3.28)  p=0.325 | 0.49 (12.36)  p=0.969 | 0.07 (0.18)  p=0.676 | 1.27 (4.90)  p=0.795 | 1.51 (3.33)  p=0.651 | 5.45 (3.01)  p=0.072 | -0.08 (0.63)  p=0.898 | -0.352 (0.535)  p=0.512 |
| cg21823999 | 4 | *GPM6A* | 1.99 (1.02)  p=0.052 | 4.01 (2.96) p=0.176 | **-4.65 (1.49)**  **p=0.002** | -16.29 (9.52) p=0.089 | **70.50 (35.47)**  **p=0.049** | 0.87 (0.49) p=0.077 | 23.68 (13.75)  p=0.087 | 11.19 (9.41) p=0.237 | 4.26 (8.84) p=0.631 | 2.65 (1.83)  p=0.148 | **-4.649 (1.485)**  **p=0.002** |
| cg23621817 | 4 | *CHRNA9* | -0.55 (1.03)  p=0.596 | -1.05 (2.95)  p=0.722 | **3.53 (1.52)**  **p=0.02** | 3.57 (9.59) p=0.710) | -**79.63 (35.08)**  **p=0.025** | **-26.18 (0.49)**  **p=0.0082** | **-31.14 (13.92)**  **p=0.027** | -12.90 (9.58) p=0.180 | -2.85 (8.81) p=0.747 | **-4.18 (1.81) p=0.022** | **3.530 (1.520)**  **p=0.021** |
| cg10296867 | 17 | *GAS7* | 0.02 (0.22)  p=0.925 | 0.12 (0.64) p=0.847 | **-0.76 (0.33)**  **p=0.024** | -1.70 (2.09) p=0.416 | 1.80 (7.79)  p=0.818 | -0.02 (0.11)  p=0.874 | 3.52 (3.08) p=0.254 | 1.57 (2.10) p=0.455 | 1.37 (1.92)  p=0.474 | 0.16 (0.40) p=0.689 | **-0.757 (0.333)**  **p=0.024** |
| cg12570712 | 19 | (intergenic) | 0.21 (0.24)  p=0.387 | 0.11 (0.71) p=0.881 | 0.34 (0.36) p=0.351 | 1.51 (2.29)  p=0.510 | -13.29 (8.49)  p=0.120 | -0.15 (0.12)  p=0.202 | **-8.76 (3.32)**  **p=0.009** | **-6.06 (2.27)**  **p=0.008** | 2.34 (2.09) p=0.263 | -0.65 (0.44) p=0.141 | 0.341 (0.364) p=0.351 |
| cg01028223 | 7 | *GET4* | -1.02 (0.58)  p=0.084 | -1.35 (1.69)  p=0.423 | 1.39 (0.88)  p=0.117 | -1.44 (5.48) p=0.793 | **-86.25 (19.42)**  **p=0.00017** | **-0.593 (0.279)**  **p=0.035** | **-18.97 (8.01) p=0.019** | -9.91 (5.50)  p=0.073 | 3.03 (5.05)  p=0.549 | -1.12 (1.05) p=0.287 | 1.387 (0.881) p=0.117 |
| cg10462017 | 7 | *MAD1L1* | 0.09 (0.65)  p=0.891 | 0.20 (1.84) p=0.912 | 0.72 (0.97)  p=0.458 | 0.45 (5.98) p=0.940 | -42.83 (22.22) p=0.056 | -0.39 (0.32)  p=0.222 | **-23.89 (8.47) p=0.005** | **-14.48 (5.80) p=0.014** | -5.23 (5.40) p=0.334 | **-3.89 (1.10) p=0.0005** | 0.719 (0.966) p=0.458 |

Bold font indicates p<0.05

**Supplementary Table 5. Associations between DNA methylation and total CVD after adjustment for traditional cardiometabolic risk factors* by median HbA1c at baseline**

| Subgroup | ID | Chr | Gene Symbol | β per 5% methylation | SE | p-value | Absolute percent change in effect size after cardiometabolic risk factor adjustment |
| --- | --- | --- | --- | --- | --- | --- | --- |
| HbA1c <8.9% | cg07147033 | 1 | *MIB2* | 0.407 | 0.170 | 0.0017 | 50.8% |
|  | cg12324048 | 3 | (intergenic) | 0.775 | 0.204 | 0.0001 | 16.1% |
|  | cg15883830 | 1 | (intergenic) | 2.057 | 0.653 | 0.001 | 35.1% |
| HbA1c ≥8.9% | cg21823999 | 4 | *GPM6A* | 4.830 | 1.372 | 0.0004 | 26.4% |
|  | cg23621817 | 4 | *CHRNA9* | -7.000 | 1.643 | 0.00002 | 18.7% |
|  | cg10296867 | 17 | *GAS7* | 1.375 | 0.311 | 0.00001 | 7.5% |
|  | cg12570712 | 19 | (intergenic) | -1.442 | 0.371 | 0.0001 | 19.7% |
|  | cg01028223 | 7 | *C7orf20 (GET4)* | -3.745 | 0.821 | 0.000005 | 7.5% |
|  | cg10462017 | 7 | *MAD1L1* | -3.510 | 0.870 | 0.00005 | 16.9% |

*Adjusted for HbA1c, body mass index, HDLc, Non-HDLc, triglycerides, systolic and diastolic blood pressure, pulse rate, albumin excretion rate, and estimated glomerular filtration rate.

**Supplementary Table 6.** Genome-wide significant (p<5.0E-8) methylation quantitative trait loci (meQTLs) for CVD-associated methylation identified in the EDC cohort

| CVD-associated CpG | meQTL accession number | meQTL Chromosome: position (hg19) | beta | Standard error | p-value | Gene | meQTL in GoDMC database (p<5x10^-8^) | Whole blood eQTL in GTeX (p<0.0005) |
| --- | --- | --- | --- | --- | --- | --- | --- | --- |
| cg14524754  cg14524754  cg14524754 | rs9909026 | 17:80925205 | -0.0221 | 0.002778 | 2.40E-14 | *B3GNTL1* | yes | no |
|  | rs59574779 | 17:80925401 | -0.02197 | 0.002775 | 3.07E-14 | *B3GNTL1* | yes | no |
|  | rs9972957 | 17:80922525 | 0.02277 | 0.003212 | 7.34E-12 | *B3GNTL1* | yes | no |
|  | rs9972880 | 17:80922965 | 0.02277 | 0.003212 | 7.34E-12 | *B3GNTL1* | yes | yes |
|  | rs7215871 | 17:80918696 | -0.0209 | 0.002967 | 9.56E-12 | *B3GNTL1* | yes | no |
|  | - | 17:80919844 | 0.02282 | 0.003265 | 1.36E-11 | *B3GNTL1* | yes | no |
|  | rs7210006 | 17:80925673 | 0.02222 | 0.003219 | 2.35E-11 | *B3GNTL1* | yes | yes |
|  | rs7223719 | 17:80925800 | 0.02222 | 0.003219 | 2.35E-11 | *B3GNTL1* | yes | yes |
|  | rs113168917 | 17:80927128 | 0.02222 | 0.003219 | 2.35E-11 | *B3GNTL1* | yes | yes |
|  | rs9903393 | 17:80926365 | 0.02198 | 0.0032 | 2.86E-11 | *B3GNTL1* | yes | no |
|  | rs66522553 | 17:80928015 | 0.02077 | 0.003169 | 1.98E-10 | *B3GNTL1* | yes | yes |
|  | rs73372864 | 17:80928396 | 0.02067 | 0.003182 | 2.78E-10 | *B3GNTL1* | yes | no |
|  | rs78072175 | 17:80928400 | 0.02067 | 0.003182 | 2.78E-10 | *B3GNTL1* | yes | no |
|  | rs9899421 | 17:80929407 | 0.02032 | 0.003133 | 2.98E-10 | *B3GNTL1* | yes | no |
|  | rs12051714 | 17:80918202 | 0.02043 | 0.003236 | 8.19E-10 | *B3GNTL1* | yes | yes |
|  | rs11650407 | 17:80931905 | -0.02509 | 0.003989 | 9.34E-10 | *B3GNTL1* | yes | yes |
|  | rs12150620 | 17:80931398 | -0.02492 | 0.003982 | 1.12E-09 | *B3GNTL1* | yes | no |
|  | rs12951871 | 17:80926089 | -0.02137 | 0.00342 | 1.18E-09 | *B3GNTL1* | yes | no |
|  | rs4986138 | 17:80926810 | -0.02137 | 0.00342 | 1.18E-09 | *B3GNTL1* | yes | no |
|  | rs12943809 | 17:80927604 | -0.02137 | 0.00342 | 1.18E-09 | *B3GNTL1* | yes | no |
|  | rs370646064 | 17:80928450 | 0.01833 | 0.002968 | 1.79E-09 | *B3GNTL1* | yes | no |
|  | rs112920095 | 17:80929458 | 0.0185 | 0.003038 | 2.93E-09 | *B3GNTL1* | yes | no |
|  | rs57424424 | 17:80930097 | -0.02255 | 0.003759 | 4.84E-09 | *B3GNTL1* | yes | no |
|  | rs75562308 | 17:80917238 | 0.01919 | 0.003199 | 4.92E-09 | *B3GNTL1* | yes | no |
|  | rs28620665 | 17:80931758 | 0.01802 | 0.003049 | 8.00E-09 | *B3GNTL1* | yes | yes |
|  | rs8075894 | 17:80853155 | -0.01699 | 0.002922 | 1.36E-08 | *TBCD* | yes | no |
|  | rs1226596224 | 17:80877005 | -0.01725 | 0.002972 | 1.44E-08 | *TBCD* | yes | no |
|  | rs186313772 | 17:80917271 | 0.01867 | 0.003239 | 1.76E-08 | *B3GNTL1* | yes | no |
|  | rs191161382 | 17:80917272 | 0.01867 | 0.003239 | 1.76E-08 | *B3GNTL1* | yes | no |
|  | rs1599898062 | 17:80917274 | 0.01867 | 0.003239 | 1.76E-08 | *B3GNTL1* | yes | no |
|  | rs8072217 | 17:80906167 | 0.01827 | 0.003175 | 1.87E-08 | *B3GNTL1* | yes | yes |
|  | rs8080526 | 17:80911679 | 0.01827 | 0.003175 | 1.87E-08 | *B3GNTL1* | yes | yes |
|  | rs2125480 | 17:80913094 | 0.01827 | 0.003175 | 1.87E-08 | *B3GNTL1* | yes | yes |
|  | rs12952956 | 17:80926421 | -0.01887 | 0.003292 | 2.11E-08 | *B3GNTL1* | yes | no |
|  | rs4986137 | 17:80926552 | -0.01887 | 0.003292 | 2.11E-08 | *B3GNTL1* | yes | yes |
|  | rs1381968095 | 17:80918383 | -0.01763 | 0.00308 | 2.21E-08 | *B3GNTL1* | yes | no |
|  | rs9906115 | 17:80791469 | 0.01909 | 0.003345 | 2.42E-08 | *TBCD; ZNF750* | yes | yes |
|  | rs6502024 | 17:80913598 | 0.01801 | 0.003167 | 2.68E-08 | *B3GNTL1* | yes | yes |
|  | rs9303016 | 17:80924707 | -0.01858 | 0.003268 | 2.70E-08 | *B3GNTL1* | yes | no |
|  | rs1551627 | 17:80875626 | -0.01819 | 0.003219 | 3.30E-08 | *TBCD* | yes | yes |
|  | rs79338499 | 17:80880236 | -0.01831 | 0.003244 | 3.37E-08 | *TBCD* | yes | no |
|  | rs2065304216 | 17:80926436 | -0.01858 | 0.0033 | 3.65E-08 | *B3GNTL1* | yes | no |
|  | rs2085615 | 17:80865406 | -0.01663 | 0.002959 | 3.84E-08 | *TBCD* | yes | no |
|  | rs2279063 | 17:80878992 | -0.01811 | 0.003223 | 3.85E-08 | *TBCD* | yes | yes |
|  | rs5822550 | 17:80855411 | -0.018 | 0.003222 | 4.61E-08 | *TBCD* | yes | no |
|  | rs4986133 | 17:80857409 | -0.018 | 0.003222 | 4.61E-08 | *TBCD* | yes | yes |
|  | rs7209726 | 17:80859872 | -0.018 | 0.003222 | 4.61E-08 | *TBCD* | yes | yes |
|  | rs12952711 | 17:80863343 | -0.018 | 0.003222 | 4.61E-08 | *TBCD* | yes | no |
|  | rs879715 | 17:80865866 | -0.018 | 0.003222 | 4.61E-08 | *TBCD* | yes | yes |
|  | rs8073910 | 17:80866427 | -0.018 | 0.003222 | 4.61E-08 | *TBCD* | yes | yes |
|  | rs2271916 | 17:80867281 | -0.018 | 0.003222 | 4.61E-08 | *TBCD* | yes | yes |
|  | rs6502012 | 17:80867499 | -0.018 | 0.003222 | 4.61E-08 | *TBCD* | yes | yes |
|  | rs6502013 | 17:80872458 | -0.018 | 0.003222 | 4.61E-08 | *TBCD* | yes | yes |
|  | rs9899957 | 17:80875429 | -0.018 | 0.003222 | 4.61E-08 | *TBCD* | yes | yes |
|  | rs8079905 | 17:80876413 | -0.018 | 0.003222 | 4.61E-08 | *TBCD* | yes | yes |
|  | rs8067859 | 17:80876866 | -0.018 | 0.003222 | 4.61E-08 | *TBCD* | yes | yes |
|  | rs8066186 | 17:80877120 | -0.018 | 0.003222 | 4.61E-08 | *TBCD* | yes | yes |
|  | rs1551626 | 17:80877432 | -0.018 | 0.003222 | 4.61E-08 | *TBCD* | yes | yes |
|  | rs1551625 | 17:80877553 | -0.018 | 0.003222 | 4.61E-08 | *TBCD* | yes | yes |
| cg07147033  cg07147033 | rs9442431 | 1:1548702 | -0.0344 | 0.004294 | 1.73E-14 | *MIB2* | yes | no |
|  | rs9442430 | 1:1548801 | -0.0344 | 0.004294 | 1.73E-14 | *MIB2* | yes | no |
|  | rs4648611 | 1:1550702 | -0.0344 | 0.004294 | 1.73E-14 | *MIB2* | yes | no |
|  | rs9726211 | 1:1551249 | -0.0344 | 0.004294 | 1.73E-14 | *MIB2* | yes | no |
|  | rs7418389 | 1:1551927 | -0.0344 | 0.004294 | 1.73E-14 | *MIB2* | yes | no |
|  | rs6604985 | 1:1552755 | -0.0344 | 0.004294 | 1.73E-14 | *MIB2* | yes | yes |
|  | rs28623434 | 1:1553593 | -0.0344 | 0.004294 | 1.73E-14 | *MIB2* | yes | yes |
|  | rs9442410 | 1:1553670 | -0.0344 | 0.004294 | 1.73E-14 | *MIB2* | yes | yes |
|  | rs4075469 | 1:1537176 | -0.0344 | 0.004297 | 1.80E-14 | *FNDC10, LOC105378586* | yes | no |
|  | rs6691649 | 1:1539582 | -0.0344 | 0.004297 | 1.80E-14 | *LOC105378586* | yes | no |
|  | rs61776787 | 1:1606571 | -0.03501 | 0.004412 | 2.92E-14 | *SLC35E2B* | no | yes |
|  | rs4612573 | 1:1546949 | -0.03392 | 0.00431 | 4.57E-14 | *n/a* | no | no |
|  | rs1186608467 | 1:1530494 | -0.03287 | 0.004253 | 1.18E-13 | *n/a* | no | no |
|  | rs28503881 | 1:1529950 | -0.03277 | 0.004301 | 2.45E-13 | *n/a* | no | no |
|  | rs4648779 | 1:1603594 | -0.03362 | 0.004777 | 1.05E-11 | *SLC35E2B* | no | yes |
|  | rs28662991 | 1:1529511 | -0.03193 | 0.004552 | 1.22E-11 | *n/a* | yes | no |
|  | rs4648771 | 1:1592630 | -0.03284 | 0.004854 | 5.70E-11 | *CDK11B, SLC35E2B* | no | no |
|  | rs9329576 | 1:1555366 | -0.0314 | 0.004778 | 1.84E-10 | *MIB2* | yes | yes |
|  | rs35051391 | 1:1532279 | -0.03014 | 0.004617 | 2.38E-10 | *n/a* | no | no |
|  | rs7532034 | 1:1603163 | -0.03314 | 0.005112 | 3.11E-10 | *SLC35E2B* | no | yes |
|  | rs146438388 | 1:1531276 | -0.02821 | 0.004368 | 3.62E-10 | *n/a* | no | no |
|  | rs6656591 | 1:1539369 | -0.02888 | 0.004563 | 7.66E-10 | *LOC105378586* | yes | yes |
|  | rs9442425 | 1:1554362 | -0.02888 | 0.004563 | 7.66E-10 | *MIB2* | yes | no |
|  | rs61774903 | 1:1543624 | -0.0281 | 0.00461 | 2.93E-09 | *n/a* | yes | yes |
|  | rs12066 | 1:1549354 | -0.0281 | 0.00461 | 2.93E-09 | *MIB2* | yes | yes |
|  | rs6671612 | 1:1545289 | -0.02687 | 0.004611 | 1.29E-08 | *n/a* | no | yes |
|  | rs1047599728 | 1:1546628 | -0.02826 | 0.004925 | 2.10E-08 | *n/a* | yes | no |
|  | rs9442431 | 1:11957778 | 0.02284 | 0.004042 | 3.34E-08 | *n/a* | no | no |
|  | rs28510084 | 1:1562437 | -0.02778 | 0.004931 | 3.67E-08 | *MIB2* | yes | no |
|  | rs11552172 | 1:1534614 | -0.02777 | 0.004938 | 3.86E-08 | *FNDC10, LOC105378586* | yes | no |
|  | rs7554569 | 1:1537437 | -0.02777 | 0.004938 | 3.86E-08 | *FNDC10, LOC105378586* | yes | no |
| cg10296867 | rs78106531 | 17:10066759 | -0.01223 | 0.002165 | 3.28E-08 | GAS7 | yes | no |
|  | rs12150284 | 17:10031090 | -0.009887 | 0.001772 | 4.77E-08 | GAS7 | yes | no |
|  | rs9913911 | 17:10031183 | -0.009887 | 0.001772 | 4.77E-08 | GAS7 | yes | no |

**Supplementary Table 7.** Gene ontology (GO) terms with p<0.05 for total CVD in EDC participants with HbA1c ≥8.9%

| **GO Term** | **Type** | **Class** | **# genes tested /total** | **Genes in Set** | **p-value** |
| --- | --- | --- | --- | --- | --- |
| GO:0005262~ calcium channel activity | MF | Transporter activity | 2/118 | *GPM6A, CHRNA9* | 0.0015 |
| GO:0099699~ integral component of synaptic membrane | CC | Synapse | 2/128 | *GPM6A, CHRNA9* | 0.0015 |

MF=molecular function, CC=cellular component.

**Supplementary Table 8. Loci included in Reactome Functional Interaction network analysis (alphabetical)**

| *APITD1* | *PTPRN2* |
| --- | --- |
| *B3GNTL1* | *SLC35E2B* |
| *CDC25B* | *TBCD* |
| *CDK11B* | *TGFB1I1* |
| *CHRNA9* | *WSCD2* |
| *FANCC* |  |
| *FCHO2* |  |
| *FNDC10* |  |
| *GAS7* |  |
| *GET4* |  |
| *GPM6A* |  |
| *GTF2IRD1* |  |
| *KIF1C* |  |
| *LINGO1* |  |
| *LYRM4* |  |
| *MAD1L1* |  |
| *MDFI* |  |
| *MIB2* |  |
| *MYL9* |  |
| *NARG2* |  |
| *NEURL1* |  |
| *POLE* |  |
| *PTGES* |  |

**Supplementary Table 9.** Significantly enriched Reactome pathways identified in functional interaction network analysis of loci containing CpGs with DNA methylation associated with CVD incidence at FDR<0.20.

| **Network Module** | **Top-level Pathway** | **Sub-pathway** | **Reaction (Stable Identifier)** | **p-value** | **FDR** | **Node** |
| --- | --- | --- | --- | --- | --- | --- |
| 1 | DNA repair | DNA repair | Fanconi Anemia Pathway (R-HSA-6783310) | 0.018 | 0.022 | *FANCC* |
| 1 | metabolism of proteins | Metabolism of proteins | Protein folding(R) | 0.030 | 0.030 | *TBCD* |
| 1 | DNA replication | DNA replication | DNA Replication Pre-Initiation (R-HSA-69002) | 0.043 | 0.043 | *POLE* |
| 1 | Cell cycle, DNA replication | DNA replication, S phase | Synthesis of DNA (R-HSA-69239 | 0.049 | 0.049 | *POLE* |
| 2 | Protein localization | Protein localization | Insertion of tail-anchored proteins into the endoplasmic reticulum membrane(R) | 0.004 | 0.015 | *GET4* |
| 2 | Sensory perception | Sensory processing of sound | Sensory processing of sound by outer hair cells of the cochlea(R) | 0.009 | 0.018 | *CHRNA9* |
| 2 | Neuronal system | Transmission across chemical synapses | Neurotransmitter receptors and postsynaptic signal transmission(R) | 0.028 | 0.028 | *CHRNA9* |
| 5 | Signal Transduction | RHO GTPase Effectors | RHO GTPases Activate ROCKs (R-HSA-5627117) | 0.005 | 0.031 | *MYL9* |
| 5 | Signal Transduction | RHO GTPase Effectors | RHO GTPases activate CIT(R) | 0.005 | 0.031 | *MYL9* |
| 5 | Signal Transduction | RHO GTPase Effectors | RHO GTPases activate PAKs(R) | 0.005 | 0.031 | *MYL9* |
| 5 | Signal Transduction | RHO GTPase Effectors | RHO GTPases activate PKNs(R) | 0.009 | 0.031 | *MYL9* |
| 5 | Muscle contraction | Muscle contraction | Smooth Muscle Contraction(R) | 0.010 | 0.031 | *MYL9* |
| 5 | Developmental biology | Axon guidance | Semaphorin interactions(R) | 0.016 | 0.031 | *MYL9* |
| 5 | Developmental biology | Axon guidance | EPH-Ephrin signaling(R) | 0.022 | 0.031 | *MYL9* |
| 5 | Signal Transduction | Death receptor signaling | p75 NTR receptor-mediated signalling (R-HSA-193704) | 0.022 | 0.031 | *LINGO1* |
| 5 | Vesicle-mediated transport | Membrane trafficking | Clathrin-mediated endocytosis (R-HSA-8856828) | 0.031 | 0.031 | *FCHO2* |


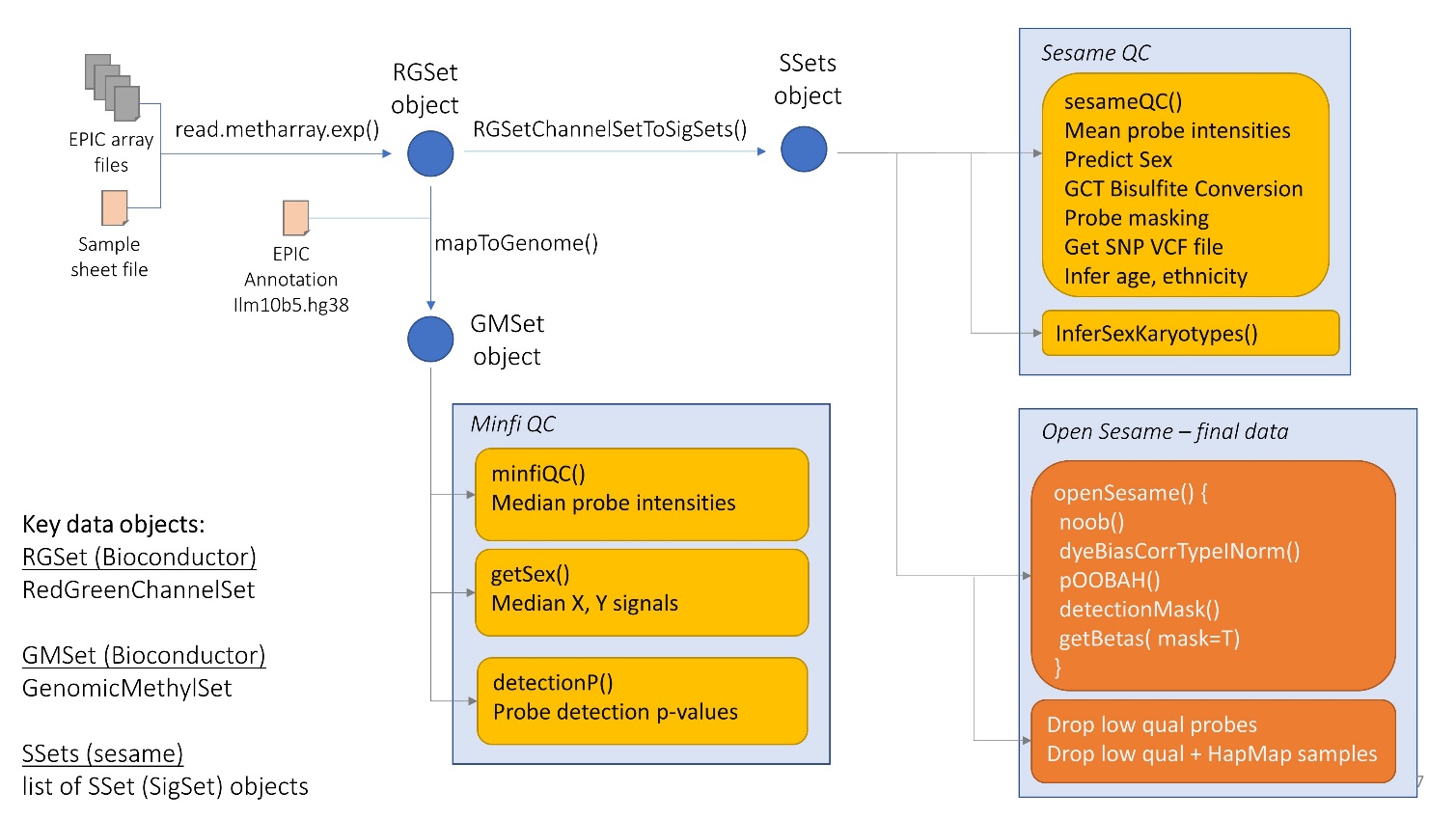


**Supplementary Figure 1.** DNA methylation quality control (QC) pipeline used in the EDC study. Of the n=11 participants whose methylation data did not pass QC, 5 were excluded by both the minfi and SeSAME pipelines, while 6 were uniquely excluded by the SeSAMe pipeline (1 due to mean probe intensities, 5 due to incomplete bisulfite conversion)


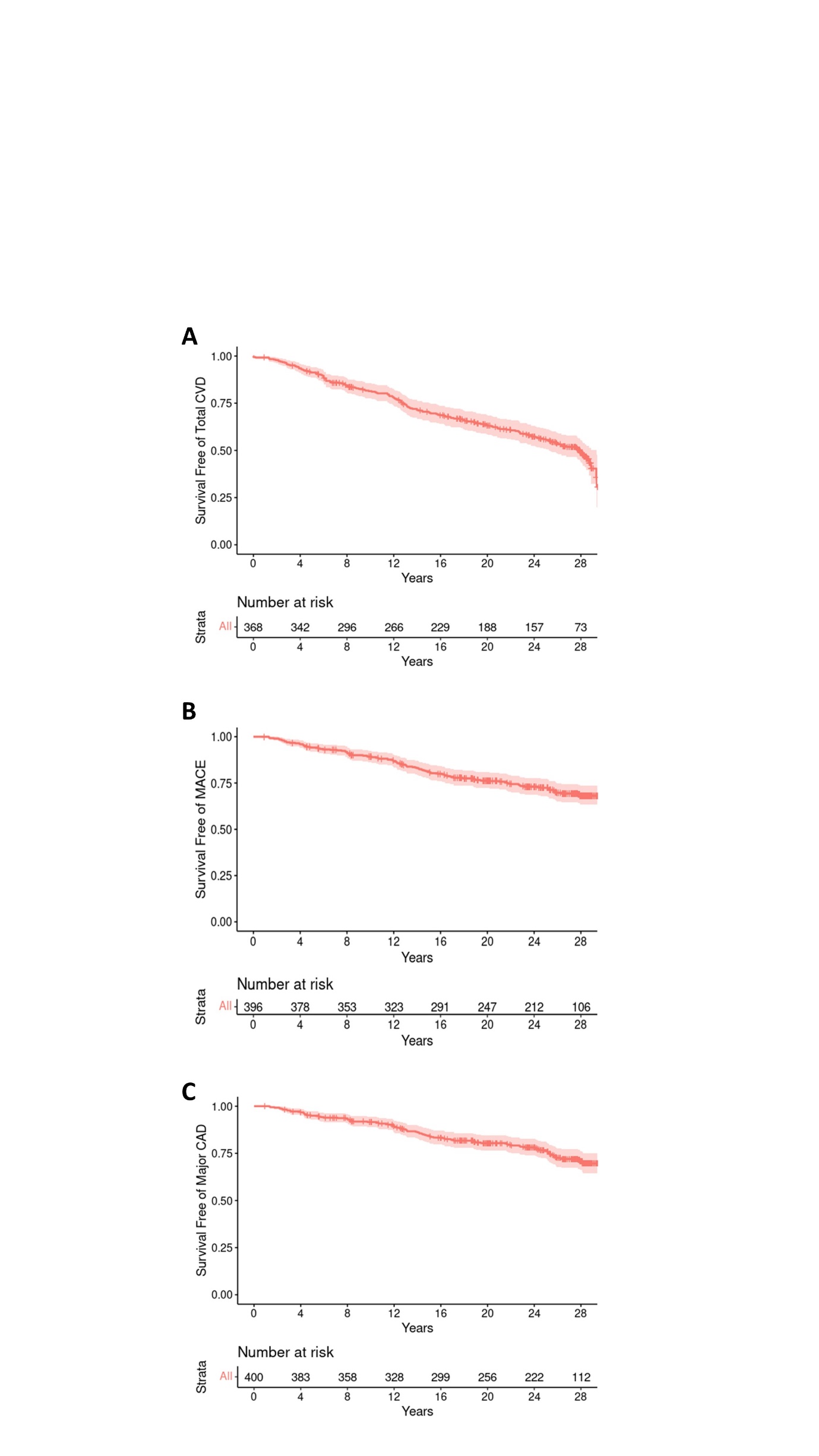


**Supplementary Figure 2**. Kaplan-Meier curves of proportion free of each CVD outcome. Panel A: Total CVD, Panel B: MACE, Panel C: Major CAD


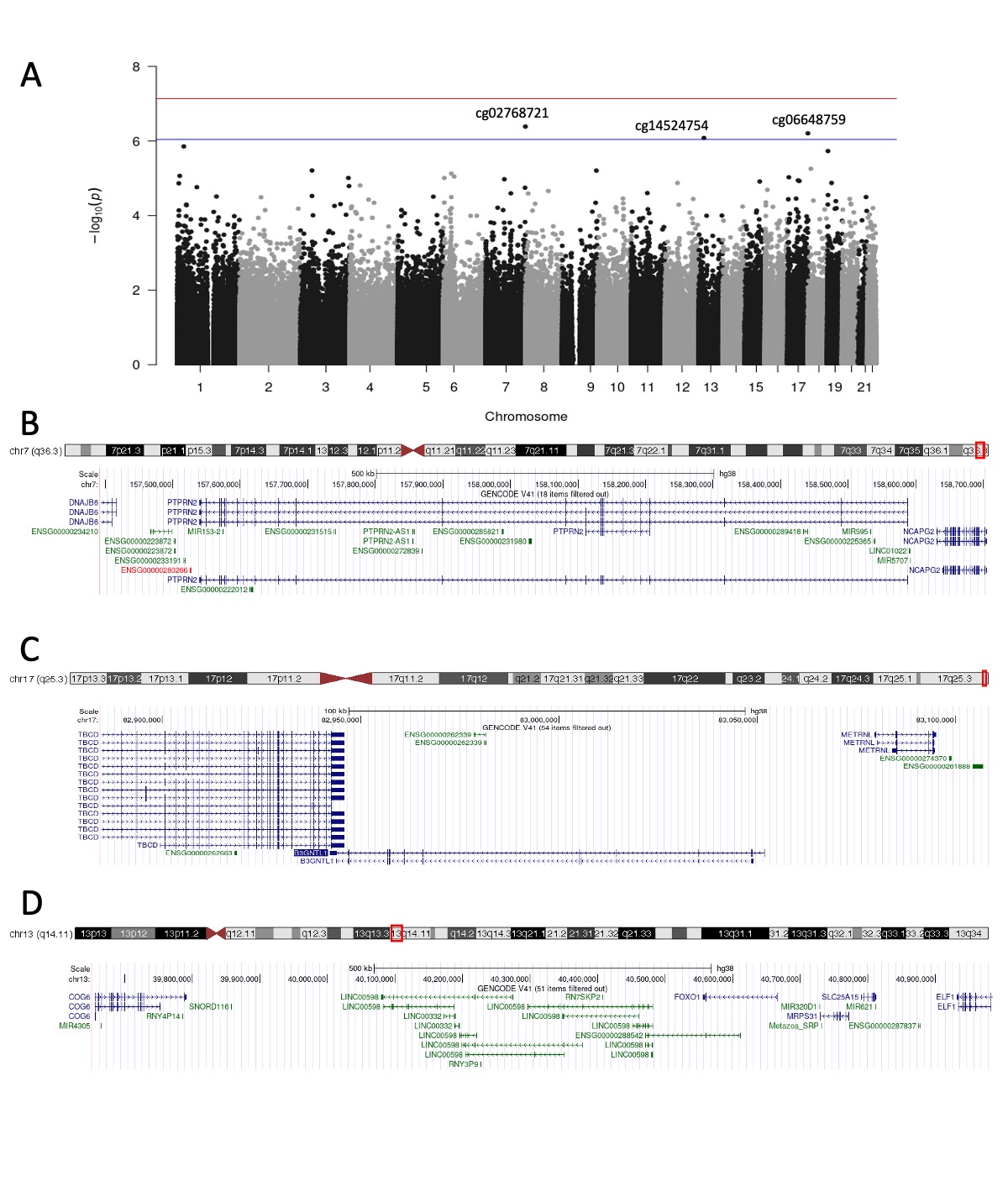


**Supplementary Figure 3.** Manhattan plot (A) for the epigenome-wide association of DNAm with time-to-CVD event after excluding probe beta outliers and gene track plots for CpGs with FDR<0.20 (B-D) in the EDC study. Red line indicates FDR<0.05, blue line indicates FDR<0.20.


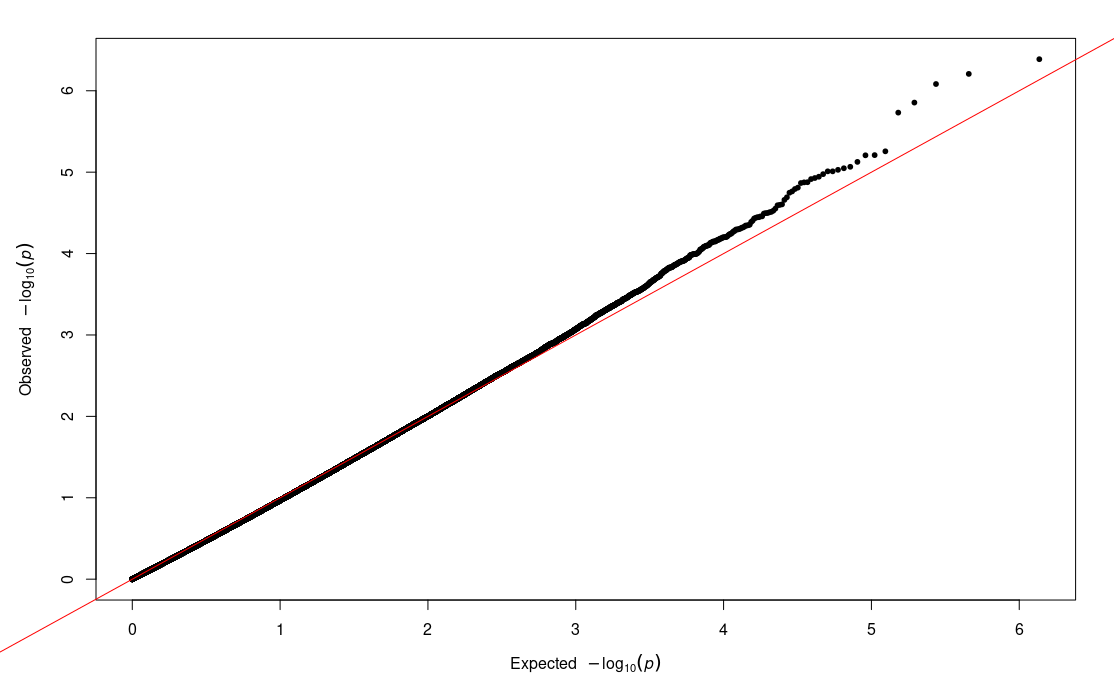


λ=0.909

**Supplementary Figure 4.** QQ plot for the epigenome-wide association of DNAme with time-to-CVD event. λ = traditional genomic inflation factor.


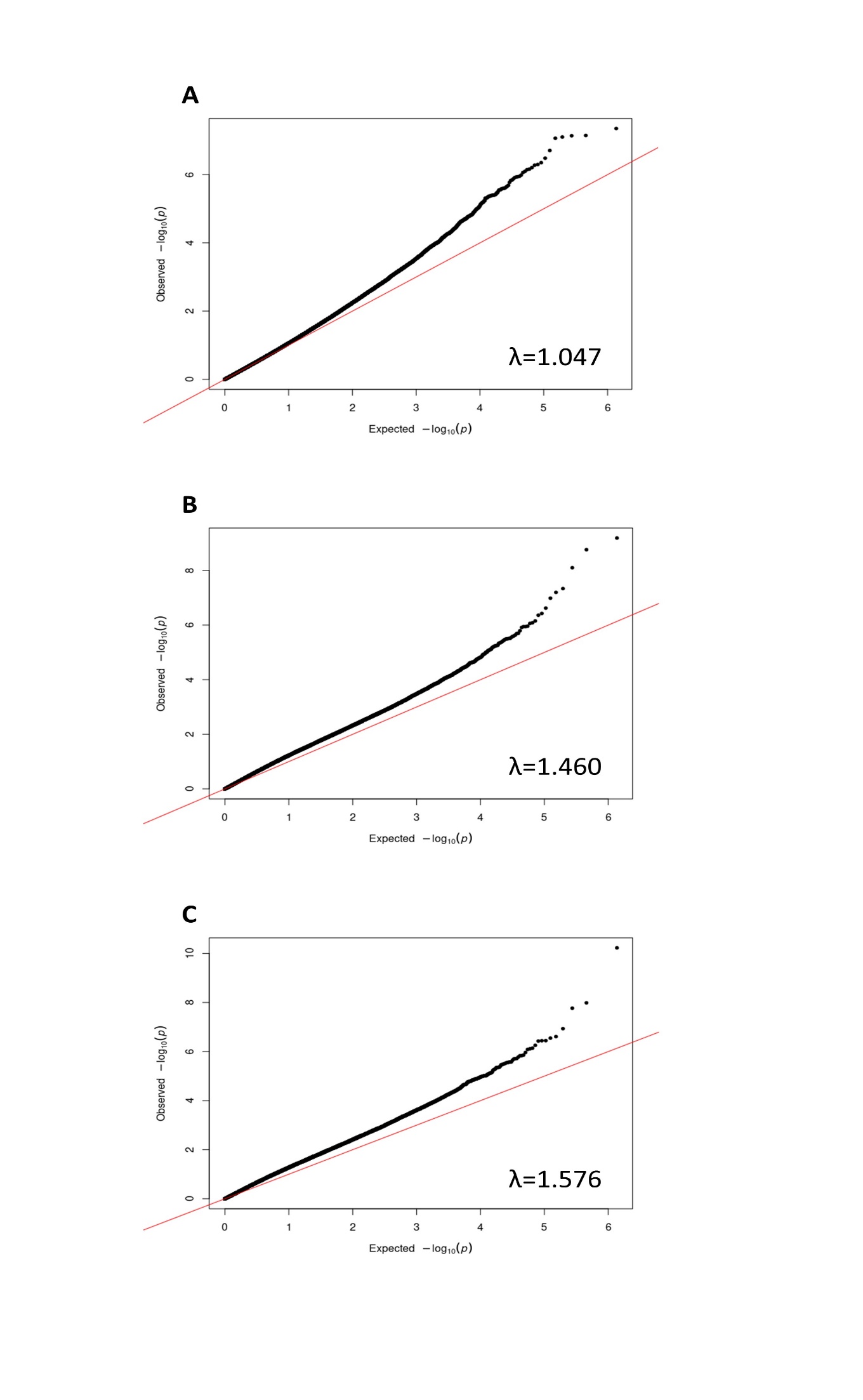


**Supplementary Figure 5.** QQ plot for the epigenome-wide association of DNAme with time-to-CVD event, all observations included (including extreme probe β values i.e., > +/-3 SD from mean). λ = traditional genomic inflation factor.
